# Assessing Genetic Variation in *Guadua angustifolia* Through RAD-Seq Analysis

**DOI:** 10.3390/ijms26125879

**Published:** 2025-06-19

**Authors:** Hair Santiago Lozano-Puentes, Lina Tarazona-Pulido, Diana López-Alvarez, Eduardo Ruiz-Sanchez, Geison M. Costa, Lucia A. Díaz-Ariza

**Affiliations:** 1 Grupo de Investigación en Agricultura Biológica, Departamento de Biología, Pontificia Universidad Javeriana, Sede Bogotá, Bogotá 110231, Colombia; h.lozano@javeriana.edu.co; 2 Grupo de Investigación Fitoquímica, Departamento de Química, Pontificia Universidad Javeriana, Sede Bogotá, Bogotá 110231, Colombia; modesticosta.g@javeriana.edu.co; 3 Grupo de Investigación en Diversidad Biológica, Departamento de Ciencias Biológicas, Facultad de Ciencias Agropecuarias, Universidad Nacional de Colombia, Sede Palmira, Palmira 763533, Colombia; 4 Departamento de Botánica y Zoología, Centro Universitario de Ciencias Biológicas y Agropecuarias, Universidad de Guadalajara, Zapopan 45200, Mexico; eduardo.ruiz@academicos.udg.mx

**Keywords:** *Guadua angustifolia*, RAD-SEQ, SNPs, genetic diversity

## Abstract

*Guadua angustifolia*, a native bamboo species of ecological and economic importance, has been widely studied in Colombia. This study focused on evaluating the genetic diversity and population structure of *G. angustifolia* from six natural populations in the Department of Nariño, Colombia, using restriction site-associated DNA sequencing (RADseq). A total of 224,996 high-quality SNPs were identified across 48 individuals. Observed heterozygosity (Ho) ranged from 0.398 in Consacá to 0.78 in Tumaco, while expected heterozygosity (He) was lower in all cases, ranging from 0.291 to 0.597. All populations exhibited negative inbreeding coefficients (FIS), from −0.316 to −0.763, indicating an excess of heterozygotes and suggesting predominantly outcrossing reproduction. Analysis of molecular variance (AMOVA) revealed that most genetic variation resides within individuals (92.54%), with low differentiation among populations (7.46%). Population structure and phylogenetic analyses identified two main genetic clusters, likely reflecting the origin of the planting material. Our results revealed that chromosomes CM070500.1, CM070502.1, CM070503.1, CM070504.1, CM070508.1, and CM070510.1 exhibited the highest SNP density, suggesting the presence of genomic regions with elevated variability. In contrast, chromosomes with lower SNP density suggested conservated genes related to Flavone Synthase II. This study is the first to evaluate genetic materials from the Department of Nariño. These findings highlight the significant genetic diversity in *G. angustifolia* and the density of SNPs, and provide suggestions for conservation planning and the development of targeted breeding programs for this non-model tropical species.

## 1. Introduction

*Guadua angustifolia* Kunth, a native bamboo species of ecological and economic importance, has been widely studied in Colombia. It is a pioneer in the structural use of bamboo and in the development of construction technologies, and a series of quality standards for its cultivation, management, and use have been developed. The area of natural and planted bamboo groves in Colombia during the last 25 years (1980–2005) has been 51,000 ha, of which 46,000 ha (90%) are natural and 5000 ha (10%) are cultivated [1]. It is distributed from the Andean region to the Amazon, and from sea level to 2200 m.a.s.l. It is located along rivers and streams in the foothills of the Andes and inter-Andean valleys. It adapts to diverse types of soils, but develops better in soils of volcanic origin. It forms dense patches in the central–western region of Colombia; however, it has been observed that small patches are formed within the Amazon rainforest. [2,3]. *G. angustifolia* is a sustainable natural resource with a high growth rate (11 to 21 cm per day), which is why several native and rural communities satisfy their basic needs with this material [4].

Bamboo has traditionally been used in Colombia, mainly for housing, handicrafts, pulp, paper, panels, boards, veneer, flooring, roofing, and fabrics, among other uses. It has been reported that approximately 90% of guadua is used in construction. Additionally, its cultivation is associated with environmental services, including ornamental and landscape beauty, thermal and acoustic regulators, flow regulators, soil protection against erosion, ecological restoration in disturbed ecosystems, and carbon sinks [5]. The use of *G. angustifolia* to produce oil, gas and charcoal (as fuel and as an excellent natural absorbent), and as a healthy plant, has also been reported [6,7].In our country, in the present year, this has led to the passing of Ministry of Agriculture and Rural Development’s resolution number 000009: “By which the Organization of the National Chain of Guadua/Bamboo and its Agroindustry is recognized under the denomination of National Council of the Productive Chain of Guadua/Bamboo and its Agroindustry”. Among the phenotypic traits, symbiotic interactions, such as mycorrhizal colonization, have recently gained relevance because of their role in nutrient uptake and plant performance [8]. Regarding their chemical composition, until 2015, flavonoids were reported to be present in canes and leaves [9,10,11]. These characteristics have been reported in materials from several municipalities in the department of Nariño, Colombia, including Tumaco, Ricaurte, La Florida, Consacá, Chachagüí, and San Lorenzo. The percentage of mycorrhizal colonization ranged from 29.6% to 96.4%, with an overall mean of 81.3 ± 15.5%. The Tumaco population showed the highest colonization levels, whereas Consacá exhibited the greatest variability among individuals [12]. Biologically important metabolites have been identified in these materials, including flavonoids, fatty acids, cinnamic acid derivatives, peptides, glycosylated lignans, alkaloids, carboxylic acids, phenols, carbohydrates, steroids, glycosylated stilbenes, and prenolipids [13]. For example, materials from San Lorenzo have phenolic compounds with high antioxidant capacity, with values of 209.23 and 144.76 μmol of Trolox per gram of extract, determined by the DPPH– and ABTS–+ methods, respectively [14]. Similarly, flavonoids and phenolic acid derivatives were identified in samples from Chachagüí and Tumaco, which showed a strong correlation with antioxidant and cytotoxic activities [15].

These findings highlight the importance of phenolic compounds, particularly flavonoids, in the biological potential of *G. angustifolia*. Among these metabolites, glycosylated flavones have been predominantly identified in the leaf extracts of this species [13,15]. In this context, flavone synthases (FNSs) constitute a key group of enzymes in flavonoid biosynthesis, as they catalyze the conversion of flavanones into flavones by introducing a double bond between the C_2_ and C_3_ positions of the central ring, allowing the formation of flavones, a subgroup of compounds with diverse biological functions [16,17,18]. In several plant species, the genes coding for these enzymes have already been described, as is the case for *Chrysanthemum morifolium* [19], *Sorghum bicolor* [20] and *Glycine max* [21]. Currently, 25 genomic sequences for the enzyme Flavone Synthase II [EC:1.14.19.76] have been deposited in the Kyoto Encyclopedia of Genes and Genomes (KEGG) for the Poaceae family, none of which correspond to bamboo species.

As technologies have advanced in the study of the chemical composition of *G. angustifolia*, so have the tools for genomic analysis of this plant. In this sense, it has been reported that *Guadua* is a tetraploid organism (2n = 4x = 46), and its genome has already been assembled [22,23]. However, despite these advances, genetic diversity studies using new-generation technologies are scarce. Among these, restriction site-associated DNA sequencing (RADSeq) has emerged. This technique provides detailed information on the genetic variability of the species. Like other reduced-representation sequencing approaches, RADseq targets a subset of the genome, thus offering advantages over whole-genome sequencing, such as a greater depth of coverage per locus (and thus greater confidence in genotype determination) and the sequencing of a larger number of samples for a given budget. Furthermore, unlike many other methods for generating genome-wide data, RADseq does not require any prior genomic information on the taxa being studied. Consequently, RADseq has become the most widely used genomic approach for the discovery and genotyping of high-throughput single-nucleotide polymorphisms (SNPs) in ecological and evolutionary studies of non-model organisms [24,25]. The use of reduced genome sequencing technologies, such as RAD-seq, has proven to be a powerful tool for resolving complex phylogenetic relationships in bamboo. In the Phyllostachys clade, this approach has allowed for remarkable phylogenetic resolution, overcoming the limitations of traditional plastid DNA-based methods and providing new insights into the systematics and evolution of temperate bamboos [26]. A time-calibrated ddRAD-based phylogenetic tree revealed that the tribe Arundinarieae underwent rapid diversification during the middle Miocene, coinciding with paleoclimatic events such as the intensification of the East Asian monsoon and the emergence of key innovations such as leptomorphic rhizomes [27]. Likewise, in the *Bambusa*–*Dendrocalamus*–*Gigantochloa* taxonomic complex (BDG complex), considered the most diverse and phylogenetically conflicting group among the paleotropical bamboos, the use of ddRAD-seq data allowed us to recover a well-resolved topology, confirming the monophyly of *Gigantochloa* and *Melocalamus*, and showing the latter as a sister group to the rest of the complex. In contrast, *Bambusa* and *Dendrocalamus* were resolved as paraphyletic, highlighting the usefulness of RAD-seq for reconstructing evolutionary histories in lineages where processes such as ancestral hybridization and polyploidy have been significantly influenced [28].

In the recent past, this technology has been applied to species of the genus *Guadua*, allowing for a higher internal phylogenetic resolution owing to its broad and efficient genomic sampling capacity, which is essential for elucidating diversification patterns in this group [29]. The implementation of high-resolution strategies, such as RAD-seq, is further justified by the levels of genetic variation documented in different species of the genus *Guadua*. For example, high values of genetic diversity have been reported in populations of *G. weberbaueri* [30], *G. trinii* [31], *G. inermis* [32], *G.* aff. *chaparensis* and *G.* aff. *lynnclarkiae*, in which high average genetic diversity was also confirmed [33]. These findings underline the importance of employing modern genomic tools to understand the evolution and genetic structures of Neotropical bamboo species.

These findings underscore the biological and chemical relevance of plant materials from the Department of Nariño and emphasize the importance of advancing their genetic characterization. The application of RADSeq enables a deeper understanding of genetic diversity and facilitates the identification of markers linked to traits such as chemical composition and plant–flavonoid interactions, supporting future breeding and conservation strategies for *G. angustifolia*. Thus, the objective of this study was to determine the genetic diversity of *G. angustifolia* populations from Colombia using RADseq. This methodology allowed for a detailed evaluation of the genetic variability among the different populations, contributing to a better understanding of the genetic structure of the species and providing key information for future conservation and genetic improvement strategies.

## 2. Results

### 2.1. RADseq Libraries and SNP Calling

Following the sequencing of RAD libraries from the 48 *G. angustifolia* samples, a total of 18 GB of raw data was generated with more that 362 million reads, and on procces_radtags with default parameters, we obtained more than 99% retained sequences encompassing over 360 million reads of 123 bp in length, and between 331 thousand and 22 million reads per sample. The dataset exhibited high-quality scores, with more than 99% of the reads achieving a Phred Q30, and an average GC content of 48% (Table S1).

In the denovo_map assembly process, the analysis started with 360,807,628 RADtags, and after analyses with cstacks and tsv2bam, 5,445,125 RAD loci were retained, with an effective mean coverage per sample of 25.0× (ranging from 5.6× to 79.9×) and a mean number of sites per locus of 123pb; furthermore, 625,391 (84.5%) diploid loci were found.

In the populations analyses using the loci retained in tsv2bam, 3,079,685 variant sites and 466,111 SNPs (Table 1) were found. Following the exclusion of SNPs with missing data through the TASSEL filtering of 80% of the SNPs, 224,996 SNPs were retained across 40 samples.

### 2.2. Genetic Diversity

The results showed variations in genetic diversity and population structure among the six populations analyzed (Tumaco, Ricaurte, La Florida, Consacá, San Lorenzo, and Chachagüí) (Table 2). Chachagüí (191,077) and San Lorenzo (185,521) have the highest number of usable loci, while La Florida has the lowest (149,633). Regarding mutation types across populations, the analysis revealed that transitions accounted for more than 50% of the identified SNPs at the population level, with a mean value of 30,223 and 19,210 transversions on all populations. Regarding the number of SNPs, Consacá had the highest value (63,667), followed by San Lorenzo (57,391), whereas Tumaco had the lowest (35,901). Regarding observed (Ho) and expected (He) heterozygosity, Tumaco has the highest Ho (0.78), while Consacá shows the lowest (0.398). He was relatively low in all populations, with Tumaco having the highest value (0.597) and La Florida the lowest (0.291). Nucleotide diversity (Pi) varied among populations, with Consacá showing the highest value (0.147 ± 0.074) and Chachagüí the lowest (0.099 ± 0.050). Theta S (ΘS) and Theta Pi (Θπ) values also reflect the differences between populations, with Consacá registering the highest values (ΘS: 19,187 ± 6798; Θπ: 23,210 ± 11,712). Similarly, the inbreeding coefficient (FIS) was negative in all populations, suggesting an excess of heterozygosity. Chachagüí (−0.763) and Ricaurte (−0.676) present the lowest values, indicating a greater deviation from the Hardy–Weinberg equilibrium.

### 2.3. Genetic Differentiation and Analysis of Molecular Variance

Table 3 presents the results of the analysis of molecular variance (AMOVA) for *G. angustifolia*. Genetic variation is decomposed into two levels: between and within populations. The variation between populations represented 7.46% of the total, with a variance component of 377.32058 (Va), whereas the variation within populations was 92.54%, with a variance component of 4683.30631 (Vb).

The FST of distance values varied among populations (Table 4), with the highest values observed between Ricaurte and Consacá (0.241) and the lowest between Consacá and Chachagüí (−0.054). Some comparisons show negative FST values, such as La Florida with Consacá (−0.044) and San Lorenzo with Chachagüí (−0.037).

### 2.4. Population Structure

**Figure 1:**
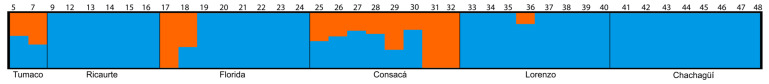
Distribution of genetic groups in *G. angustifolia* populations determined by ADMIXTURE. Clustering of 40 individuals based on the admixture estimate for K = 2. Each bar indicates an individual, while the colors correspond to belonging to a particular group; the number of groups is determined by the K value, where blue corresponds to K1, and orange is K2.

Analysis of the ADMIXTURE population structure of the species evaluated revealed similar patterns to the genetic diversity analyses (Figure 1), showing that a two-cluster model (K = 2) was the most likely structure for the dataset. The Tumaco, Consacá, and San Lorenzo populations showed a combination of both colors, indicating the presence of more than one genetic group within them. In contrast, Ricaurte, La Florida, and Chachagüí populations were predominantly blue, indicating greater genetic homogeneity within these populations.

**Figure 2:**
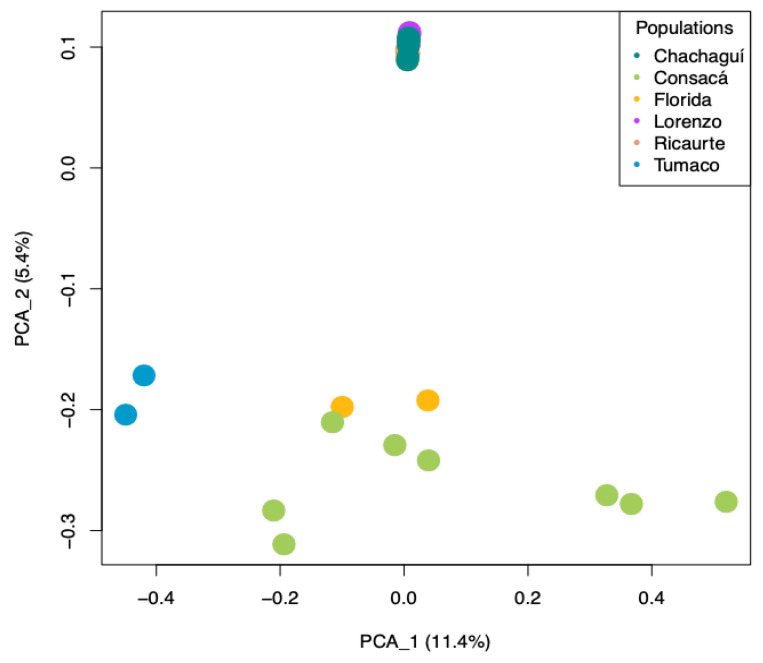
Principal component analysis (PCA) plot showing genetic relationships among the six natural populations of *G. angustifolia*. The dots represent the populations of origin: blue for Tumaco, green for Consacá, yellow for La Florida, red for Ricaurte, purple for San Lorenzo, and aquamarine for Chachagüí.

**Figure 3:**
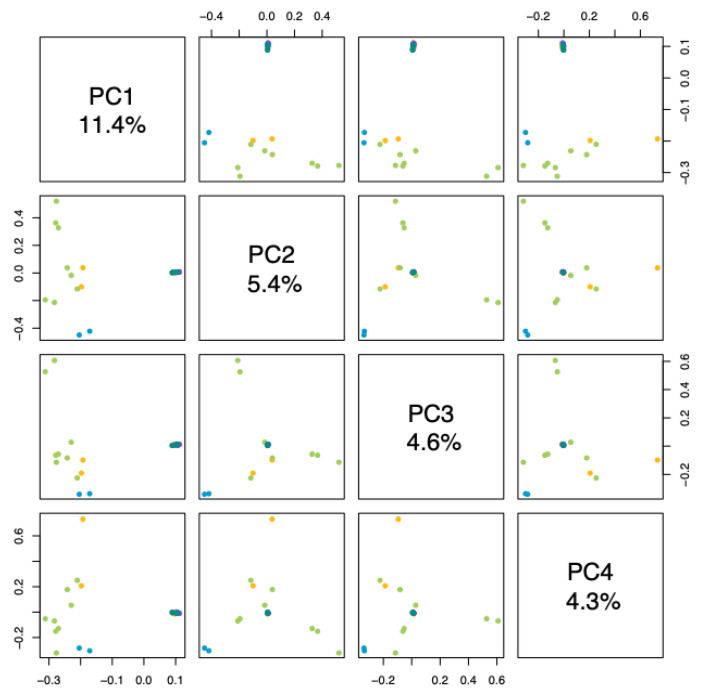
Analysis of the main components and four components of *G. angustifolia*. The dots represent the population of origin: blue for Tumaco, green for Consacá, yellow for La Florida, purple for San Lorenzo and aquamarine for Chachagüí.

Principal component analysis (PCA) provides a pattern of the samples. Samples that are close to each other indicate their similarities, whereas those that are far from each other indicate their differences. We observed that the variables studied explained 16.08% of the groupings (Figure 2). The materials from Tumaco and Consacá, and some individuals from La Florida were grouped in principal component 1, while the other materials were grouped at a single point in principal component 2, indicating a significant differentiation between these two groups. Similarly, in the analysis of the principal components and four components of *G. angustifolia* (Figure 3), the materials from Chachagüí, Ricaurte, and San Lorenzo and the remaining one from La Florida continued to group closely together, indicating genetic similarity between them.

**Figure 4:**
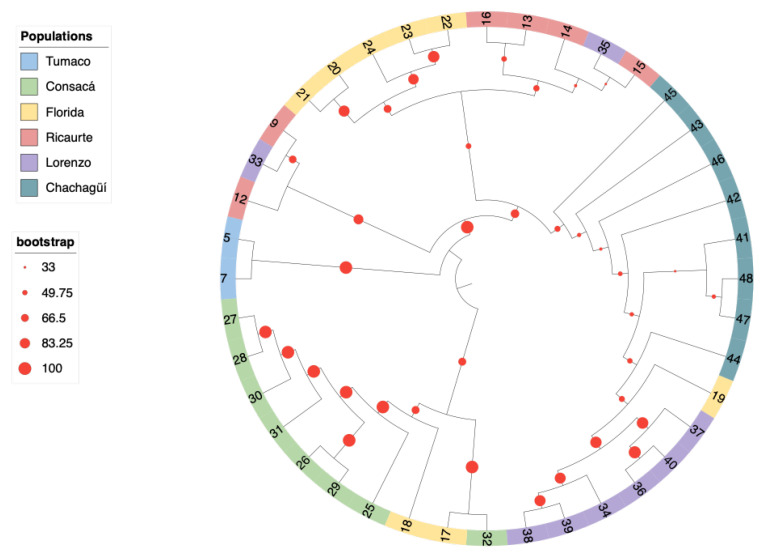
Phylogenetic tree based on the natural *G. angustifolia* population phylogenetic tree inferred by maximum likelihood from the analysis of 40 individuals. The branches are colored according to the population of origin: blue for Tumaco, green for Consacá, yellow for La Florida, red for Ricaurte, purple for San Lorenzo, and aquamarine for Chachagüí.

The maximum likelihood phylogeny showed the same trend as the other analyses; the optimal model given by jModelTest was GTR + G with a gamma value of 0.903, revealing two clades, each with a bootstrap robustness value of 100 (Figure 4). These results are consistent with those obtained from the Identical by Descent (IBD) analysis (Table S2). For instance, individuals 17 (La Florida) and 32 (Consacá) showed an IBD value of 0.44, indicating that they were genetically identical. Similarly, individuals 21 (La Florida) and 15 (Ricaurte) had values of 0.41. In a different clade, individuals 27 and 28 from Consacá also shared an IBD value of 0.41.

**Figure 5:**
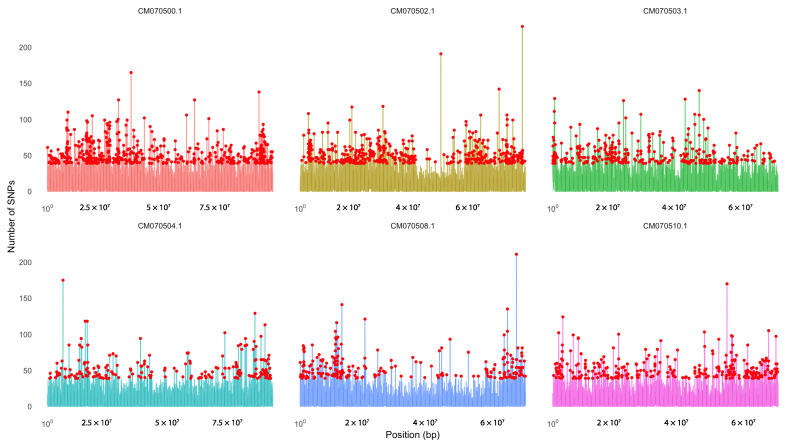
SNP density across the six chromosomes with the highest concentration of variants in *Guadua angustifolia*. A high SNP density was observed in chromosomes CM070500.1, CM070502.1, CM070503.1, CM070504.1, CM070508.1, and CM070510.1, identified as genomic hotspots based on RAD-seq data. SNP density was calculated using 10 kb sliding windows, revealing specific regions with notable variant accumulation, potentially associated with adaptive or diversification processes.

SNP hotspot analysis was performed on the RAD-seq data obtained for *Guadua angustifolia*, which allowed the identification of genomic regions with a high density of variants. Figure 5 shows the six chromosomes that had the highest number of SNPs, specifically CM070500.1, CM070502.1, CM070503.1, CM070504.1, CM070508.1, and CM070508.1, which stand out as potential regions of high genetic variability. Figure S1 shows the total distribution of SNPs along all chromosomes of the reference genome (GCA_036346375.1), which revealed heterogeneity in the distribution of these variants.

## 3. Discussion

Over the years, different molecular techniques have been used to explore the genetic diversity of *G. angustifiolia* owing to the socioeconomic importance of this plant in our country. From the analysis of amplified fragment length polymorphism (AFLP) in different species of the genus *Guadua*, clear genetic differentiation between the different species was observed. However, low diversity has been observed among accessions of *G. angustifolia* [34]. An approach using random amplified polymorphic DNA analysis in *G. angustifolia* indicated high genetic diversity among and within 12 biotypes of the species [35]. The study of microsatellite markers in *G. angustifolia* showed high genetic diversity (He = 0.54) and a high Ho value (0.68), indicating an excess of heterozygous individuals [20]. Another study, with random amplified microsatellites (RAMs) in *G. angustifolia*, reported He = 0.31, which indicates high allele diversity and that the selected materials correspond to different genotypes and not genetically uniform clones [36]. In this study, the results were similar to those described above, with the observed heterozygosity being greater than the expected heterozygosity. An excess of heterozygotes in a population means that the proportion of individuals with different alleles at a genetic locus is higher than expected under the Hardy–Weinberg equilibrium.

This excess of heterozygotes in the population indicates the avoidance of inbreeding, as there may be factors that favor cross-pollination. The flower of *G. angustifolia* is complete, has anthers and a female structure in the same floret, while the pollen is viable, but the stigma is located in a way that prevents self-fertilization; it could be considered that there is self-incompatibility, but more detailed studies should be carried out to determine the exact genetic cause or causes that originate from the excess of heterozygotes. An inflorescence is a set of pseudospikelets, and each pseudospikelet is formed by multiple florets. Inside a floret, the gynoecium is formed by a style that ends in three feathery stigmas and by the androcecium formed by six stamens whose filaments are very elongated and hang below the stigmas, making self-fertilization difficult. This allogamy favors the formation of heterozygotes [37,38]. In this study, the FIS fixation index for all populations was additionally found (values between −0.7 and −0.3). This index quantifies the decrease in heterozygosity in a natural population as a consequence of inbreeding [39]. Heterozygote formation was supported by negative values. However, the fact that *G. angustifolia* is a tetraploid organism (2n = 4x = 46) supports the idea that there is greater genetic diversity among samples, since a tetraploid population of size N has a total of 4N chromosomal copies at an autosomal locus, compared to diploid populations with only 2N copies. Consequently, with a mutation rate of µ, the number of mutations per generation was twice as high in the tetraploid population: 4Nµ versus 2Nµ [40].

The AMOVA analysis, which showed a variance of 7.46% between populations, indicated low genetic differentiation. This suggests that, although the populations are somewhat differentiated, most of the alleles are still shared among them, possibly because of gene flow between populations. Within populations, genetic variation (92.54%) occurred between individuals within each population owing to the algal nature of the species, indicating high individual heterozygosity, a common pattern in species with cross-propagation or clonal propagation with genetic admixture, as frequently occurs in tropical bamboo of the genus *Guadua* [33,41,42]. In *G. angustifolia*, a high gene flow has been previously reported, which explains results such as low Fst and low distances between groups. Any level of gene flow between populations prevents complete differentiation between them and, therefore, maintains relative homogeneity among groups. These results coincide with those reported by Posso [43], where from the set of results obtained, the evaluated groups of *G. angustifolia* in the Colombian “Eje Cafetero” region could be considered a single population that exchanges a good proportion of genetic information, and the presence of moderate population structuring can be explained by a selection process that favors the prevalence of an excess of heterozygotes. This is confirmed by the results obtained by Muñoz-Florez [36], who, when comparing materials from Colombia and Peru, observed a clear difference between them that was attributed to geographical distance, i.e., the more distant the populations, the greater the genetic difference.

Here, we can observe that the materials from Consacá and Tumaco are differentiated from those from the other municipalities. The lack of exact correspondence between genetic and geographic groups in some cases may be associated with the relationship of the species with the pre-Columbian inhabitants who were able to transport material from one region to another, and in some cases, with the migration of strains or seeds by water currents, birds, and other animals [38]. The materials from Consacá could have genetic information corresponding to materials from Quindío, since on the farm where the sampling was carried out, the owners informed us that the material was a donation of plants from this municipality. In Tumaco, there is no information regarding the origin of the “guaduales”. In contrast, in the other municipalities that belong to the second genetic group, the information that was collected was that they are “guaduales” established naturally in this department. This suggests that the differences between the groups analyzed are determined by the origin of the parental material of each population; however, a comparative study including materials from Quindío could confirm this hypothesis. Finally, the phylogenetic tree allowed for the grouping of materials with greater precision, revealing the similarity between the different materials evaluated. This is different from the results obtained by Marulanda [34], who studied different species of *Guadua*, where the accessions of *G. angustifolia* included samples from Ricaurte Nariño, Quindío, Putumayo, and others, which were grouped together.

Our results revealed that chromosomes CM070500.1, CM070502.1, CM070503.1, CM070504.1, CM070508.1, and CM070510.1 exhibited the highest SNP density, suggesting the presence of genomic regions with elevated variability [44]. This may contribute to the differentiation of the two populations described previously, as the high variability observed in these chromosomes suggests the possible action of diversifying selection, reflecting distinct adaptive processes between groups. For instance, materials from Tumaco, located 7 m above sea level, and those from Consacá, where soils were waterlogged, may respond to specific environmental pressures. However, further functional exploration of the genes located on these chromosomes is needed to confirm this hypothesis and better understand the mechanisms underlying this genetic differentiation. In contrast, chromosomes CM070506.1, CM070507.1, and CM070521.1 displayed lower SNP density, indicating they may be more conserved. This finding is particularly relevant because three of the 25 genes identified by homology as encoding the enzyme Flavone Synthase II [EC:1.14.19.76] in other Poaceae species through BLAST (https://blast.ncbi.nlm.nih.gov/Blast.cgi, accessed on 13 October 2024) analysis were located on these low-variability chromosomes. Specifically, CM070506.1 showed 92% query coverage and 83.26% identity with *Oryza brachyantha*, CM070507.1 showed 91% coverage and 83.14% identity with *Lolium perenne*, and CM070521.1 showed 74% coverage and 75.80% identity with *Panicum hallii*. The overlap between these conserved regions and the putative FNSII genes suggests that these loci may be under purifying selection, maintaining sequence integrity across evolutionary time due to their potential functional relevance in flavonoid biosynthesis, particularly in response to stress conditions [45]. The observed low genetic variability may thus reflect evolutionary constraints aimed at avoiding deleterious mutations in functionally essential genes [46].

From a conservation perspective, identifying conserved genomic regions linked to key metabolic functions is crucial. These loci may represent core components of the adaptive potential of the species, and their preservation should be prioritized in future genetic conservation and management programs for *G. angustifolia*. Identifying conserved genomic regions under purifying selection offers valuable insight into essential biological functions that support environmental resilience and the genetic basis of traits that contribute to environmental resilience, supporting reported strategies for preserving genetic diversity and functional integrity across natural populations [47,48,49]. The SNPs detected can even be used as molecular markers to monitor genetic diversity in conservation programs, ensuring genetic representativeness in each strategy implemented. On the other hand, the characterization of regions of high variability could be expanded, which could also be included in germplasm banks, ensuring adequate coverage of diversity. Exploring this SNP variability could help explain the variation in chemical composition [50], which, in turn, may be related to differences in the biological activity [51] of *Guadua* species. This understanding is key for decision-making processes aimed at conserving valuable genetic material from distinct populations.

## 4. Materials and Methods

### 4.1. Plant Material

Six natural “guaduales” belonging to *Guadua angustifolia* collected from different municipalities of the Department of Nariño, Colombia, were evaluated, as described in Table 5. The reference species were deposited in the Herbarium of the Pontificia Universidad Javeriana (HPUJ) and were determined by Néstor García and Ximena Londoño. Between three and five leaf samples were collected from each individual, ensuring that they were young leaves or new shoots in good condition without signs of predation, fungi, or pathogens. The samples were preserved in paper envelopes with silica gel to avoid decomposition or fungal growth.

### 4.2. DNA Extraction and RADseq Library Preparation

DNA extraction and RADseq library preparation followed the methodology of Tarazona-Pulido [52]. In summary, the genomic DNA (gDNA) was isolated from 100 mg of fresh leaf tissue using Wizard^®^ Genomic DNA Purification (PROMEGA, Madison, WI, USA) (Promega Corporation, Madison, WI, USA) following the manufacturer’s protocol. DNA concentrations were measured by Qubit 4™ DNA assays, and a Kit Qubit™ 1X dsDNA high-sensitivity (HS) fluorometer (Thermo Fisher Scientific Inc., Waltham, MA, USA). The quality of gDNA samples was evaluated using a Berthold Technologies Colibri microvolume spectrometer and by 2% agarose gel stained with GelRed DNA stain using the ENDURO™ Gel XL Electrophoresis System; RADseq libraries were constructed using a protocol adapted from Baird [53], through Floragenex Inc. (Beaverton, OR, USA), using the restriction enzyme PstI. The samples were sequenced on an Illumina^®^ NovaSeq 6000 platform to produce 100 bp single-end reads.

### 4.3. Bioinformatic Analyses

#### 4.3.1. Demultiplexing the Data and SNP Calling

The quality of raw reads was assessed using FastQC v 0.12.1 [54]. The STACKS v 2.66 software pipeline [55] was employed for SNP calling. Then, the cstacks script generated the catalog of consensus loci by merging the alleles from each population, and comparisons were made between samples from the created catalog using the sstacks tool. Next, gstacks identified and genotyped the SNPs within the metapopulation under the variant calling model and genotype likelihoods [56], with a threshold alpha ranging between 0.01 and 0.05 for each locus. The “populations” script then generated the VCF (Variant Calling File) output file used for genetic and structure analysis.

Additionally, TASSEL v5 [57] filtered the VCF file of denovo_map, where a locus was required to be present on 80% of the individuals.

#### 4.3.2. Genetic Diversity Analyses

The genetic diversity of the natural populations was assessed by estimating observed heterozygosity (Ho), expected heterozygosity (He), transition and transversion numbers, nucleotide compositions and diversity (Pi), and the number of polymorphic sites using the Tajima D test. We evaluated the molecular indices Theta S (ΘS) and Theta Pi (Θπ), which represent the distribution of variation within or between populations based on polymorphic sites, values of the inbreeding coefficient (FIS), and the HW test in Arlequin v 3.5.2.2 [58]; we also analyzed the gene flow level among all populations with the Nm parameter using equation Nm  =  (1 − Fst)/(4Fst).

#### 4.3.3. Population Structure and Phylogeny

Three distinct methods were used to ascertain the genetic structure of the populations. First, ADMIXTURE v 1.3.0 [58] was employed, utilizing the maximum likelihood algorithm to explore the genetic structure of the data. Ten independent runs were performed for each K value, from 1 to 5. To visualize the optimal number of delta K values and the model of population structure, the Q and P files were used as input in the software STRUCTURE SELECTOR (http://lmme.ac.cn/StructureSelector/, accessed on 13 October 2024) [59], along with a population map. Second, we performed a principal component analysis (PCA) using the SNPrelate v 1.34.1. Reference [60] package and ggplot2 v 3.4.2 [61] in R 4.3.2 (R Core Team, 2021) to extract the synthetic variables to represent major genetic variations. Thirdly, a maximum likelihood (ML) phylogenetic inference was performed using IQ-TREE v 2.1.4 [62] with 150,000 bootstrap replicates. The tree was visualized using iTOL v 5 [63] and the substitution model was determined using the software jModelTest v 2.1.10 [64], employing the Bayesian model selection criterion with support from 24 models provided by default. An AMOVA test was conducted considering Wright’s population structure statistics in Arlequin [58].

#### 4.3.4. Identical by Descent (IBD)

To assess the genetic relationships among individuals in the study population, we conducted a relatedness and identity by descent (IBD) analysis based on SNP data using the SNPRelate package (version 1.30.1) in R (version 4.2.3). The IBD analysis was performed using the maximum likelihood estimation (MLE). This method estimates the probabilities of sharing 0, 1, or 2 alleles identical by descent (IBD) between pairs of individuals, under the assumptions of the Hardy–Weinberg equilibrium and linkage equilibrium.

#### 4.3.5. SNP’s Hotspot Analysis

To identify genomic hotspot regions in *Guadua angustifolia*, we used the reference genome available in the NCBI database (accession: GCA_036346375.1). The genome was indexed using BWA, and raw sequencing reads were aligned with the *bwa mem* algorithm [65]. The resulting alignments were processed with SAMtools to generate sorted BAM files [66]. SNP calling and initial filtering were performed using FreeBayes, and the variant files were further refined with BCFtools [67]. To quantify SNP density, we used BEDTools to count the number of SNPs within non-overlapping 10 kb genomic windows across the entire genome. The resulting SNP density data were analyzed and visualized using the tidyverse and ggplot2 packages in R, allowing the identification of regions with high and low SNP accumulation, which we interpreted as potential genomic hotspots and conserved regions, respectively.

## 5. Conclusions

This study is the first to evaluate genetic materials from the Department of Nariño, underlining the importance of continuing with the characterization of these resources. Recently, *Guadua angustifolia* has attracted increasing interest because of its biological potential, especially its chemical composition, which is rich in phenolic compounds. The samples evaluated included those from Tumaco, Ricaurte, San Lorenzo, La Florida, Chachagüí and Consacá. In addition, antioxidant and cytotoxic activities have been reported in samples from Chachagüí and Tumaco, highlighting the relevance of these materials in biotechnological and pharmacological applications. The analysis of genetic diversity in these materials is crucial for identifying the most relevant characteristics that could be exploited in the improvement of the species, both in terms of yield and bioactive properties. The appropriate selection of genetic materials not only optimizes the use of resources but also ensures their sustainability and conservation, allowing the responsible and efficient use of *G. angustifolia* in future research and commercial applications. Notably, our findings revealed the presence of both highly variable and conserved genomic regions. The conserved regions, particularly those where key biosynthetic genes such as Flavone Synthase II are located, may be under purifying selection, highlighting their potential functional importance and evolutionary constraint. Preserving this genomic integrity is critical for maintaining essential metabolic functions, such as flavonoid biosynthesis, which contribute to the plant’s defense and adaptation.

## Figures and Tables

**Table 1:** Population summary after assembly in STACKS.

Population	Total No. of Sites	Variants	Total No. of SNPs
Tumaco	72,539.17	417,031	44,855
Ricaurte	146,263.60	509,148	66,946
La Florida	148,561.66	533,485	76,134
Consacá	237,316.337	573,023	105,470
Sa Lorenzo	123,021.76	501,280	80,997
Chachagüí	188,048.81	545,718	91,709

**Table 2:** Summarized genetic diversity statistics.

Population	Tumaco	Ricaurte	La Florida	Consacá	San Lorenzo	Chachagüí
No. of usable loci	177,310	153,650	149,633	157,202	185,521	191,077
No. Ts	22,602	23,456	33,062	38,740	34,598	28,884
No. Tv	13,299	14,239	20,619	24,927	22,793	19,384
SNPs	35,901	37,695	53,681	63,667	57,391	48,268
Ho	0.78	0.589	0.407	0.398	0.507	0.585
He	0.597	0.375	0.291	0.313	0.313	0.348
Pi	0.139 ± 0.090	0.104 ± 0.053	0.119 ± 0.060	0.147 ± 0.074	0.110 ± 0.055	0.099 ± 0.050
Theta S (Θ_S)_	19,582 ± 10,513	12,482 ± 4768	16,177 ± 5731	19,187 ± 6798	17,295 ± 6128	14,546 ± 5153
Theha Pi (Θ_π_)	24,664 ± 16,097	16,013 ± 8285	17,877 ± 9021	23,210 ± 11,712	20,564 ± 10,377	19,091 ± 9633
FIS	−0.609	−0.676	−0.436	−0.316	−0.661	−0.763

No. Ts, transitions sites; No. Tv, transversions sites; SNPs, number of polymorphic sites; Ho, observed heterozygosity; He, expected heterozygosity; Pi, nucleotidic diversity; ΘS, Theta S; Θπ, Theta Pi; FIS, inbreeding coefficient.

**Table 3:** Analyses of molecular variance (AMOVA) between populations for *G. angustifolia*.

Source of Variation	d.f	Sum of Squares	Variance Components	Percentage Variation
Among populations groups	5	48,017.83	377.32058 Va	7.46
Within populations groups	74	346,564.67	4683.30631 Vb	92.54

**Table 4:** Distance method: pairwise differences for *G. angustifolia* FST populations were above the diagonal, while significance values (*p* < 0.001)) were below the diagonal.

Population	Tumaco	Ricaurte	La Florida	Consacá	San Lorenzo	Chachagüí
Tumaco	0	0.082	0.168	0.175	0.098	0.156
Ricaurte	*	0	0.218	0.241	0.146	0.218
La Florida	*	*	0	−0.044	−0.026	−0.051
Consacá	*	*	*	0	−0.028	−0.054
Sa Lorenzo	*	-	*	*	0	−0.037
Chachagüí	*	*	-	*	-	0

Asterisk (*) means significantly different; hyphen minus (-) means not significant.

**Table 5:** *Guadua angustifolia* sample collection.

Collection Place	Samples Code	Latitude	Longitude	Elevation (m)	Voucher Number
Tumaco	5; 7	1.619639	−78.789861	7	30717 HPUJ
Ricaurte	9; 12 to 16	1.232028	−78.030306	1.100	30718 HPUJ
La Florida	17 to 24	1.300000	−77.403800	2.142	30719 HPUJ
Consacá	25 to 32	1.254000	−77.487972	1.598	30720 HPUJ
San Lorenzo	33 to 40	1.596778	−77.203472	1.712	30721 HPUJ
Chachagūí	41 to 48	1.374861	−77.281861	1.865	30722 HPUJ

## Data Availability

The raw RAD sequence data for the 48 samples of *G. angustifolia* have been deposited in the National Center for Biotechnology Information (NCBI) Bioproject under the accession number PRJNA1251128 and the samples among SAMN47998348 to SAMN47998395 number.

## Supplementary Materials

The supporting information can be downloaded at https://www.mdpi.com/article/10.3390/ijms26125879/s1.

## References

[1] Londoño X. (2011). El bambú en Colombia. Biot. Veg..

[2] Londoño X. (1990). Aspectos sobre la distribución y la ecología de los bambúes de Colombia (Poaceae: Bambusoideae). Caldasia.

[3] Londoño X. (2021). Diversidad de Bambúes. Diversidad de Bambúes en los Municipios de Florencia, Albania, San José de Fragua y Cartagena del Chaira del Departamento del Caquetá y Municipio La Macarena del Departamento del Meta—Colombia.

[4] Londoño X., Camayo G.C., Riaño N.M., López Y. (2002). Characterization of the anatomy of *Guadua angustifolia* (Poaceae: Bambusoideae) culms. Bamboo Science and Culture: J. Amer. Bamboo Soc..

[5] Villanueva F.P., Cóndor J.P., Alca A.M. (2014). Experiencias sobre la silvicultura y usos del bambú en Colombia. Xilema.

[6] Gutiérrez G.O., de Lira Fuentes R.C. (2020). Elaboración de biocarbón para el aprovechamiento de residuos proveniente de las podas de bambú (*Guadua angustifolia*). Rev. Mex. Agroecosistemas.

[7] Cadena J.F.A., Valverde B.R., Íñiguez J.C., Barrera L.C., Sánchez J.P.J., Carrera D.C.M. (2019). Posibilidades del bambú (*Guadua angustifolia* Kunth) para la alimentación humana en la Sierra Nororiental de Puebla, México. Nova Sci..

[8] Avendaño-Uribe B.E., Díaz L.A., Castillo-Brieva D. (2012). Model for mycorrhizal, soil and climate conditions analysis on productivity in Colombian bamboo forest. X Congreso Latinoamericano de Dinámica de Sistemas.

[9] Mosquera Martnez O.M., Gonzÿlez Cadavid L.M. (2012). Caracterización fitoquímica de los extractos de acetona y contenido de lignina en culmos de *Guadua angustifolia*. Recur. Nat. Ambiente.

[10] Durango Álvarez E.S., Gallardo Cabrera C., Contreras Contreras A. (2015). Estudios para el aprovechamiento potencial de hojas de *Guadua angustifolia* Kunth (Poaceae), para el sector cosmético. Rev. Cuba. Farmacia.

[11] Mosquera O.M., González L.M., Cortés Y.J., Camargo J.C. (2015). Caracterización fitoquímica, determinación del contenido de lignina y la actividad antioxidante de los culmos de *Guadua angustifolia* Kunth. Rev. Fac. Cienc. Básicas.

[12] Sánchez-Matiz J.J. (2024). Relación de la micorrización, la diversidad de Glomeromycota y las propiedades fisicoquímicas del suelo en poblaciones naturales de *Guadua angustifolia* Kunth. Bachelor’s Thesis.

[13] Chitiva L.C., Lozano-Puentes H.S., Londoño X., Leão T.F., Cala M.P., Ruiz-Sanchez E., Costa G.M. (2023). Untargeted metabolomics approach and molecular networking analysis reveal changes in chemical composition under the influence of altitudinal variation in bamboo species. Front. Mol. Biosci..

[14] Lozano-Puentes H.S., Sánchez-Matiz J.J., Ruiz-Sanchez E., Costa G.M., Díaz-Ariza L.A. (2023). *Guadua angustifolia* Kunth leaves as a source for bioactive phenolic compounds: Optimization of ultrasound-assisted extraction using response surface methodology and antioxidant activities. Heliyon.

[15] Chitiva L.C., Rezende-Teixeira P., Leão T.F., Lozano-Puentes H.S., Londoño X., Díaz-Ariza L.A., Castro-Gamboa I. (2024). Metabolomic Profiling of *Guadua* Species and Its Correlation with Antioxidant and Cytotoxic Activities. ACS Omega.

[16] Li H., Dong Li D., Yang Z., Zeng Q., Luo Y., He N. (2020). Flavones produced by mulberry flavone synthase type I constitute a defense line against the ultraviolet-B stress. Plants.

[17] Zheng J., Zhao C., Liao Z., Liu X., Gong Q., Zhou C., Sun C. (2023). Functional characterization of two flavone synthase II members in citrus. Hortic. Res..

[18] Jiang Y., Ji X., Duan L., Ye P., Yang J., Zhan R., Ma D. (2019). Gene mining and identification of a flavone synthase II involved in flavones biosynthesis by transcriptomic analysis and targeted flavonoid profiling in *Chrysanthemum indicum* L.. Ind. Crops Prod..

[19] Wang Y., Zhou L.J., Wang Y., Liu S., Geng Z., Song A., Chen F. (2021). Functional identification of a flavone synthase and a flavonol synthase genes affecting flower color formation in *Chrysanthemum morifolium*. Plant Physiol. Biochem..

[20] Du Y., Chu H., Wang M., Chu I.K., Lo C. (2010). Identification of flavone phytoalexins and a pathogen-inducible flavone synthase II gene (SbFNSII) in sorghum. J. Exp. Bot..

[21] Jiang Y.N., Wang B., Li H., Yao L.M., Wu T.L. (2010). Flavonoid production is effectively regulated by RNAi interference of two flavone synthase genes from *Glycine max*. J. Plant Biol..

[22] Guo Z.H., Ma P.F., Yang G.Q., Hu J.Y., Liu Y.L., Xia E.H., Li D.Z. (2019). Genome sequences provide insights into the reticulate origin and unique traits of woody bamboos. Mol. Plant.

[23] Ma P.F., Liu Y.L., Guo C., Jin G., Guo Z.H., Mao L., Li D.Z. (2024). Genome assemblies of 11 bamboo species highlight diversification induced by dynamic subgenome dominance. Nat. Genet..

[24] Andrews K.R., Good J.M., Miller M.R., Luikart G., Hohenlohe P.A. (2016). Harnessing the power of RADseq for ecological and evolutionary genomics. Nat. Rev. Genet..

[25] Lowry D.B., Hoban S., Kelley J.L., Lotterhos K.E., Reed L.K., Antolin M.F., Storfer A. (2017). Breaking RAD: An evaluation of the utility of restriction site-associated DNA sequencing for genome scans of adaptation. Molecular Ecology Resources.

[26] Wang X., Ye X., Zhao L., Li D., Guo Z., Zhuang H. (2017). Genome-wide RAD sequencing data provide unprecedented resolution of the phylogeny of temperate bamboos (Poaceae: Bambusoideae). Sci. Rep..

[27] Guo C., Ma P.F., Yang G.Q., Ye X.Y., Guo Y., Liu J.X., Li D.Z. (2021). Parallel ddRAD and genome skimming analyses reveal a radiative and reticulate evolutionary history of the temperate bamboos. Syst. Biol..

[28] Liu J.X., Zhou M.Y., Yang G.Q., Zhang Y.X., Ma P.F., Guo C., Li D.Z. (2020). ddRAD analyses reveal a credible phylogenetic relationship of the four main genera of Bambusa-Dendrocalamus-Gigantochloa complex (Poaceae: Bambusoideae). Mol. Phylogenetics Evol..

[29] Ruiz-Sanchez E., Maya-Lastra C., Perez-Garcia M.D.L.L., Garcia-Martinez M.A. (2025). Phylogenomics and biogeography of Guadua: Insights into a neotropical woody bamboo genus. Am. J. Bot..

[30] da Silva Almeida Leal G., Leal F.A., Gomes H.T., de Souza A.M., Ribeiro S.C., Scherwinski-Pereira J.E. (2021). Structure and genetic diversity of natural populations of *Guadua weberbaueri* in the southwestern Amazon, Brazil. J. For. Res..

[31] Perez-Garcia L., Pérez-Alquicira J., Rico Y., Vargas-Ponce O., Montti L., Ruiz-Sanchez E. (2025). Despite forest fragmentation, river connectivity maintains gene flow and diversity in *Guadua trinii*, a woody bamboo of the Atlantic Forest in Argentina. Hydrobiologia.

[32] Perez-Alquicira J., Aguilera-Lopez S., Rico Y., Ruiz-Sanchez E. (2021). A population genetics study of three native Mexican woody bamboo species of Guadua (Poaceae: Bambusoideae: Bambuseae: Guaduinae) using nuclear microsatellite markers. Bot. Sci..

[33] Silva S.M., Martins K., Costa F.H., Campos T.D., Scherwinski-Pereira J.E. (2020). Genetic structure and diversity of native Guadua species (Poaceae: Bambusoideae) in natural populations of the Brazilian Amazon rainforest. An. Acad. Bras. Ciências.

[34] Marulanda M.L., Márquez P., Londoño X. (2002). AFLP analysis of *Guadua angustifolia* (Poaceae: Bambusoideae) in Columbia with emphasis on the coffee region. J. Am. Bamboo Soc..

[35] Potosí C.T., Vallejo F.A., Palacio J.D. (2006). Estimación mediante RAPD’s de la diversidad genética en *Guadua* en el departamento del Cauca, Colombia. Acta Agronómica.

[36] Rugeles-Silva P.A., Posso-Terranova A.M., Londoño X., Barrera-Marín N., Muñoz-Flórez J.E. (2012). Caracterización molecular de *Guadua angustifolia* Kunth mediante marcadores moleculares RAMs. Acta Agronómica.

[37] Muñóz Florez J.E., Londoño X., Rugeles P., Posso A.M., Alirio Vallejo F. (2010). Diversidad y estructura genética de *Guadua angustifolia* en la Ecorregión Cafetera colombiana. Recur. Nat. Ambiente.

[38] Muñoz Flórez J.E. (2011). Diversidad Genética, Estructura Poblacional y Selección de Clones Superiores de *Guadua angustifolia* Kunth en la Eco-Región Cafetera de Colombia. Ph.D. Thesis.

[39] Caujapé J. (2006). Brújula para Botánicos Desorientados en la Genética de Poblaciones.

[40] Meirmans P.G., Liu S., van Tienderen P.H. (2018). The analysis of polyploid genetic data. J. Hered..

[41] Yeasmin L., Ali M.N., Gantait S., Chakraborty S. (2015). Bamboo: An overview on its genetic diversity and characterization. 3 Biotech..

[42] Oumer O.A., Dagne K., Feyissa T., Tesfaye K., Durai J., Hyder M.Z. (2020). Genetic diversity, population structure, and gene flow analysis of lowland bamboo [*Oxytenanthera abyssinica* (A. Rich.) Munro] in Ethiopia. Ecol. Evol..

[43] Posso A. (2011). Diversidad Genética y Estructura Poblacional de *Guadua angustifolia* Kunth en el eje Cafetero Colombiano. Master’s Thesis.

[44] Nosil P., Funk D.J., Ortiz-Barrientos D. (2009). Divergent selection and heterogeneous genomic divergence. Mol. Ecol..

[45] Wu J., Lv S., Zhao L., Gao T., Yu C., Hu J., Ma F. (2023). Advances in the study of the function and mechanism of the action of flavonoids in plants under environmental stresses. Planta.

[46] Rensing S.A. (2014). Gene duplication as a driver of plant morphogenetic evolution. Curr. Opin. Plant Biol..

[47] Chung M.Y., Merilä J., Li J., Mao K., López-Pujol J., Tsumura Y., Chung M.G. (2023). Neutral and adaptive genetic diversity in plants: An overview. Front. Ecol. Evol..

[48] Pang F., Niu J., Solanki M.K., Nosheen S., Liu Z., Wang Z. (2022). PHD-finger family genes in wheat (*Triticum aestivum* L.): Evolutionary conservatism, functional diversification, and active expression in abiotic stress. Front. Plant Sci..

[49] Wang G., Ives A.R., Zhu H., Tan Y., Chen S.-C., Yang J., Wang B. (2022). Phylogenetic conservatism explains why plants are more likely to produce fleshy fruits in the tropics. Ecology.

[50] Puentes H.S.L., Costa G.M., Ariza L.A.D. (2022). PPV-4 Determinación de la composición de flavonoides de hojas de *G. angustifolia* Kunth en guaduales naturales del departamento de Nariño. Rev. Prod. Nat..

[51] Kazlauckas J., Guaratini M.T.G., Moreno P.R.H. (2025). Exploring the Discrepancies in the Biological Activities of Extracts From *Guadua angustifolia* Var. Bicolor Londoño Collected in Two Different Sites. Chem. Biodivers..

[52] Tarazona-Pulido L., Rugeles-Silva P.A., Cardona Tobar K.M., Díaz-Ariza L.A., Muñoz Florez J.E., López-Álvarez D. (2024). Approach of Genetic Diversity of *Lippia alba* (Mill) and *Petiveria alliacea* L.: Medicinal Plants of Colombia. Plant Mol. Biol..

[53] Baird N.A., Etter P.D., Atwood T.S., Currey M.C., Shiver A.L., Lewis Z.A., Johnson E.A. (2008). Rapid SNP discovery and genetic mapping using sequenced RAD markers. PLoS ONE.

[54] Wingett S.W., Andrews S. (2018). FastQ Screen: A tool for multi-genome mapping and quality control. F1000Research.

[55] Catchen J.M., Amores A., Hohenlohe P., Cresko W., Postlethwait J.H. (2011). Stacks: Building and genotyping loci de novo from short-read sequences. G3 Genes Genomes Genet..

[56] Maruki T., Lynch M. (2017). Genotype calling from population-genomic sequencing data. G3 Genes Genomes Genet..

[57] Bradbury P.J., Zhang Z., Kroon D.E., Casstevens T.M., Ramdoss Y., Buckler E.S. (2007). TASSEL: Software for association mapping of complex traits in diverse samples. Bioinformatics.

[58] Excoffier L., Lischer H.E. (2010). Arlequin suite ver 3.5: A new series of programs to perform population genetics analyses under Linux and Windows. Mol. Ecol. Resour..

[59] Li Y.L., Liu J.X. (2018). StructureSelector: A web-based software to select and visualize the optimal number of clusters using multiple methods. Mol. Ecol. Resour..

[60] Zheng X., Levine D., Shen J., Gogarten S.M., Laurie C., Weir B.S. (2012). A high-performance computing toolset for relatedness and principal component analysis of SNP data. Bioinformatics.

[61] Wickham H. (2009). Manipulating data. Ggplot2: Elegant Graphics for Data Analysis.

[62] Minh B.Q., Schmidt H.A., Chernomor O., Schrempf D., Woodhams M.D., Von Haeseler A., Lanfear R. (2020). IQ-TREE 2: New models and efficient methods for phylogenetic inference in the genomic era. Mol. Biol. Evol..

[63] Letunic I., Bork P. (2021). Interactive Tree Of Life (iTOL) v5: An online tool for phylogenetic tree display and annotation. Nucleic Acids Res..

[64] Darriba D., Taboada G.L., Doallo R., Posada D. (2012). jModelTest 2: More models, new heuristics and parallel computing. Nat. Methods.

[65] Clausen P.T., Aarestrup F.M., Lund O. (2018). Rapid and precise alignment of raw reads against redundant databases with KMA. BMC Bioinform..

[66] Xu G., Deng N., Zhao Z., Judeh T., Flemington E., Zhu D. (2011). SAMMate: A GUI tool for processing short read alignments in SAM/BAM format. Source Code Biol. Med..

[67] Garrison E., Marth G. (2012). Haplotype-based variant detection from short-read sequencing. arXiv.

