# Supplementary material for: Assessing Genetic Variation in Guadua angustifolia Through RAD-Seq Analysis
Source: Int J Mol Sci. 2025 Jun 19;26(12):5879. doi: 10.3390/ijms26125879 (PMC12192596; doi:10.3390/ijms26125879)
Supplement: Supplementary file 1 [file ijms-26-05879-s001.zip › ijms-3621207-supplementary.pdf]

Assessing genetic variation in *Guadua angustifolia* through RAD-Seq Analysis

Hair Santiago Lozano-Puentes [1,2], Lina Tarazona-Pulido [3], Diana López-Alvarez [3], Eduardo Ruiz-Sanchez [4], Geison M. Costa [2] and Lucia A Díaz-Ariza[1]*

Supplementary Material

Table S1. Stacks processing

ID	Filename	Barcode	process_radtags			fastqc		
			Total RADtag	Low Quality	Retained	Total sequence	Total bases Mbp	%GC
gu1	1	TCCGGAGCGCTGCAG	1.302.676	5.530	1.297.146	1.297.146	123	49
gu2	2	CTAACACGGCTGCAG	1.996.522	8.261	1.988.261	1.988.261	123	48
gu3	3	AGCTTCGATTTGCAG	835.910	3.591	832.319	832.319	123	48
gu4	4	TCGCCGCAATTGCAG	355.425	1.586	353.839	353.839	123	48
gu5	5	TCAGTTCCGGTGCAG	5.383.418	22.611	5.360.807	5.360.807	123	48
gu6	6	CGGAAGTGAGTGCAG	1.246.200	5.250	1.240.950	1.240.950	123	48
gu7	7	GTTGCTAGACTGCAG	2.821.937	11.918	2.810.019	2.810.019	123	48
gu8	8	AATAGATTCATGCAG	997.929	4.264	993.665	993.665	123	48
gu9	9	AGCTGATACATGCAG	3.248.450	13.644	3.234.806	3.234.806	123	48
gu10	10	ATCAGTAGAATGCAG	332.495	1.366	331.129	331.129	123	49
gu11	11	TCGTCTTAGTTGCAG	1.682.592	6.917	1.675.675	1.675.675	123	48
gu12	12	GCTCAGCCAGTGCAG	3.538.785	14.718	3.524.067	3.524.067	123	50
gu13	13	CGGCTACTTCTGCAG	13.143.213	55.257	13.087.956	13.087.956	123	49
gu14	14	CAAGCCGGTTTGCAG	13.599.539	57.146	13.542.393	13.542.393	123	49
gu15	15	TTGCGCAAGCTGCAG	9.962.027	41.918	9.920.109	9.920.109	123	48
gu16	16	TACGATGGAGTGCAG	4.036.236	17.234	4.019.002	4.019.002	123	49
gu17	17	GCAATATACATGCAG	15.968.512	67.581	15.900.931	15.900.931	123	47
gu18	18	AAGAATTCGGTGCAG	3.407.569	14.470	3.393.099	3.393.099	123	47
gu19	19	TCGGCAGTCGTGCAG	2.960.105	12.557	2.947.548	2.947.548	123	48
gu20	20	AGTTCCATTGTGCAG	6.742.532	28.248	6.714.284	6.714.284	123	48
gu21	21	TTCTTGCGCTTGCAG	20.308.623	85.379	20.223.244	20.223.244	123	48
gu22	22	AGCAATCTAATGCAG	3.663.007	15.477	3.647.530	3.647.530	123	49
gu23	23	GAATTGTCGCTGCAG	8.207.862	34.183	8.173.679	8.173.679	123	48
gu24	24	CTTCGACATATGCAG	19.910.335	83.964	19.826.371	19.826.371	123	48
gu25	25	GAGATATGGTTGCAG	14.670.812	61.474	14.609.338	14.609.338	123	48

gu26	26	CTCCTTGGAGTGCAG	6.041.633	25.737	6.015.896	6.015.896	123	49
gu27	27	GTGTCTCTTGTGCAG	8.643.446	36.652	8.606.794	8.606.794	123	48
gu28	28	TGCAGTTATCTGCAG	12.413.433	52.549	12.360.884	12.360.884	123	48
gu29	29	TTCTGGAATATGCAG	4.794.666	20.227	4.774.439	4.774.439	123	52
gu30	30	ACGCAACACATGCAG	4.384.936	18.392	4.366.544	4.366.544	123	51
gu31	31	ACTGCCTCAATGCAG	9.290.918	39.643	9.251.275	9.251.275	123	47
gu32	32	ACATCAATATTGCAG	13.237.791	56.034	13.181.757	13.181.757	123	47
gu33	33	CCTCTTATCATGCAG	3.640.027	15.150	3.624.877	3.624.877	123	48
gu34	34	TATCGTTAGTTGCAG	5.595.147	23.586	5.571.561	5.571.561	123	47
gu35	35	TAGTGCGGTCTGCAG	6.890.813	28.900	6.861.913	6.861.913	123	48
gu36	36	GGCCGGTAACTGCAG	7.997.368	33.613	7.963.755	7.963.755	123	48
gu37	37	AGGAACCTCGTGCAG	7.321.951	30.889	7.291.062	7.291.062	123	48
gu38	38	TTATCCGTAGTGCAG	6.073.781	25.806	6.047.975	6.047.975	123	48
gu39	39	CGCTATACGGTGCAG	6.895.750	29.059	6.866.691	6.866.691	123	48
gu40	40	CACGCAACGATGCAG	8.026.611	33.571	7.993.040	7.993.040	123	49
gu41	41	TGTCCTAGGATGCAG	8.241.560	34.600	8.206.960	8.206.960	123	48
gu42	42	ATCCGTCTACTGCAG	3.742.266	16.026	3.726.240	3.726.240	123	49
gu43	43	GGACTCACGGTGCAG	10.577.007	44.783	10.532.224	10.532.224	123	49
gu44	44	GCGTCCTGCCTGCAG	7.374.447	31.089	7.343.358	7.343.358	123	48
gu45	45	ACTTGACCGGTGCAG	11.667.376	49.295	11.618.081	11.618.081	123	49
gu46	46	AATGGTGACTTGCAG	18.884.319	79.358	18.804.961	18.804.961	123	48
gu47	47	CTAACAGTATTGCAG	22.500.256	94.541	22.405.715	22.405.715	123	47
gu48	48	TCATAGGCTATGCAG	7.776.124	32.665	7.743.459	7.743.459	123	48
			362334337	1526709	360807628	360807628		48

Table S2. Kinship relationship of the individuals of *G. angustifolia*.

ID1	ID2	k0	k1	niter	kinship	Kinship Relationship
5	9	1	0	234	0	unrelated individuals
5	12	1	0	230	0	unrelated individuals
5	13	1	0	299	0	unrelated individuals
5	14	1	0	296	0	unrelated individuals
5	15	1	0	258	0	unrelated individuals
5	16	1	0	234	0	unrelated individuals
5	19	1	0	229	0	unrelated individuals
5	20	1	0	245	0	unrelated individuals
5	21	1	0	423	0	unrelated individuals
5	22	1	0	230	0	unrelated individuals
5	23	1	0	262	0	unrelated individuals
5	24	1	0	423	0	unrelated individuals
5	33	1	0	232	0	unrelated individuals
5	34	1	0	240	0	unrelated individuals

5	35	1	0	238	0	unrelated individuals
5	36	1	0	253	0	unrelated individuals
5	37	1	0	253	0	unrelated individuals
5	38	1	0	237	0	unrelated individuals
5	39	1	0	245	0	unrelated individuals
5	40	1	0	235	0	unrelated individuals
5	41	1	0	253	0	unrelated individuals
5	42	1	0	223	0	unrelated individuals
5	43	1	0	265	0	unrelated individuals
5	44	1	0	248	0	unrelated individuals
5	45	1	0	270	0	unrelated individuals
5	46	1	0	410	0	unrelated individuals
5	47	1	0	493	0	unrelated individuals
5	48	1	0	247	0	unrelated individuals
7	9	1	0	219	0	unrelated individuals
7	12	1	0	207	0	unrelated individuals
7	13	1	0	254	0	unrelated individuals
7	14	1	0	253	0	unrelated individuals
7	15	1	0	236	0	unrelated individuals
7	16	1	0	220	0	unrelated individuals
7	19	1	0	216	0	unrelated individuals
7	20	1	0	231	0	unrelated individuals
7	21	1	0	279	0	unrelated individuals
7	22	1	0	217	0	unrelated individuals
7	23	1	0	237	0	unrelated individuals
7	24	1	0	287	0	unrelated individuals
7	33	1	0	219	0	unrelated individuals
7	34	1	0	229	0	unrelated individuals
7	35	1	0	225	0	unrelated individuals
7	36	1	0	234	0	unrelated individuals
7	37	1	0	236	0	unrelated individuals
7	38	1	0	225	0	unrelated individuals
7	39	1	0	232	0	unrelated individuals
7	40	1	0	220	0	unrelated individuals
7	41	1	0	236	0	unrelated individuals
7	42	1	0	210	0	unrelated individuals
7	43	1	0	241	0	unrelated individuals
7	44	1	0	230	0	unrelated individuals
7	45	1	0	243	0	unrelated individuals
7	46	1	0	279	0	unrelated individuals
7	47	1	0	290	0	unrelated individuals

7	48	1	0	231	0	unrelated individuals
9	17	1	0	255	0	unrelated individuals
9	18	1	0	229	0	unrelated individuals
9	25	1	0	236	0	unrelated individuals
9	26	1	0	191	0	unrelated individuals
9	27	1	0	206	0	unrelated individuals
9	28	1	0	211	0	unrelated individuals
9	29	1	0	182	0	unrelated individuals
9	30	1	0	188	0	unrelated individuals
9	31	1	0	218	0	unrelated individuals
9	32	1	0	242	0	unrelated individuals
12	17	1	0	248	0	unrelated individuals
12	18	1	0	220	0	unrelated individuals
12	25	1	0	233	0	unrelated individuals
12	26	1	0	189	0	unrelated individuals
12	27	1	0	207	0	unrelated individuals
12	28	1	0	211	0	unrelated individuals
12	29	1	0	182	0	unrelated individuals
12	30	1	0	188	0	unrelated individuals
12	31	1	0	218	0	unrelated individuals
12	32	1	0	239	0	unrelated individuals
13	17	1	0	354	0	unrelated individuals
13	18	1	0	264	0	unrelated individuals
13	25	1	0	276	0	unrelated individuals
13	26	1	0	216	0	unrelated individuals
13	27	1	0	237	0	unrelated individuals
13	28	1	0	247	0	unrelated individuals
13	29	1	0	209	0	unrelated individuals
13	30	1	0	212	0	unrelated individuals
13	31	1	0	258	0	unrelated individuals
13	32	1	0	307	0	unrelated individuals
14	17	1	0	363	0	unrelated individuals
14	18	1	0	262	0	unrelated individuals
14	25	1	0	275	0	unrelated individuals
14	26	1	0	215	0	unrelated individuals
14	27	1	0	236	0	unrelated individuals
14	28	1	0	244	0	unrelated individuals
14	29	1	0	208	0	unrelated individuals
14	30	1	0	212	0	unrelated individuals
14	31	1	0	256	0	unrelated individuals
14	32	1	0	306	0	unrelated individuals

15	17	1	0	297	0	unrelated individuals
15	18	1	0	248	0	unrelated individuals
15	25	1	0	257	0	unrelated individuals
15	26	1	0	205	0	unrelated individuals
15	27	1	0	225	0	unrelated individuals
15	28	1	0	231	0	unrelated individuals
15	29	1	0	197	0	unrelated individuals
15	30	1	0	202	0	unrelated individuals
15	31	1	0	243	0	unrelated individuals
15	32	1	0	271	0	unrelated individuals
16	17	1	0	253	0	unrelated individuals
16	18	1	0	224	0	unrelated individuals
16	25	1	0	233	0	unrelated individuals
16	26	1	0	190	0	unrelated individuals
16	27	1	0	206	0	unrelated individuals
16	28	1	0	212	0	unrelated individuals
16	29	1	0	182	0	unrelated individuals
16	30	1	0	190	0	unrelated individuals
16	31	1	0	221	0	unrelated individuals
16	32	1	0	240	0	unrelated individuals
17	19	1	0	252	0	unrelated individuals
17	20	1	0	272	0	unrelated individuals
17	21	1	0	611	0	unrelated individuals
17	22	1	0	256	0	unrelated individuals
17	23	1	0	292	0	unrelated individuals
17	24	1	0	597	0	unrelated individuals
17	33	1	0	262	0	unrelated individuals
17	34	1	0	272	0	unrelated individuals
17	35	1	0	267	0	unrelated individuals
17	36	1	0	293	0	unrelated individuals
17	37	1	0	291	0	unrelated individuals
17	38	1	0	264	0	unrelated individuals
17	39	1	0	280	0	unrelated individuals
17	40	1	0	261	0	unrelated individuals
17	41	1	0	284	0	unrelated individuals
17	42	1	0	248	0	unrelated individuals
17	43	1	0	306	0	unrelated individuals
17	44	1	0	280	0	unrelated individuals
17	45	1	0	313	0	unrelated individuals
17	46	1	0	489	0	unrelated individuals
17	47	1	0	727	0	unrelated individuals

17	48	1	0	281	0	unrelated individuals
18	19	1	0	225	0	unrelated individuals
18	20	1	0	237	0	unrelated individuals
18	21	1	0	279	0	unrelated individuals
18	22	1	0	224	0	unrelated individuals
18	23	1	0	251	0	unrelated individuals
18	24	1	0	293	0	unrelated individuals
18	33	1	0	237	0	unrelated individuals
18	34	1	0	237	0	unrelated individuals
18	35	1	0	231	0	unrelated individuals
18	36	1	0	244	0	unrelated individuals
18	37	1	0	249	0	unrelated individuals
18	38	1	0	232	0	unrelated individuals
18	39	1	0	239	0	unrelated individuals
18	40	1	0	229	0	unrelated individuals
18	41	1	0	242	0	unrelated individuals
18	42	1	0	220	0	unrelated individuals
18	43	1	0	253	0	unrelated individuals
18	44	1	0	242	0	unrelated individuals
18	45	1	0	256	0	unrelated individuals
18	46	1	0	281	0	unrelated individuals
18	47	1	0	294	0	unrelated individuals
18	48	1	0	240	0	unrelated individuals
19	25	1	0	231	0	unrelated individuals
19	26	1	0	193	0	unrelated individuals
19	27	1	0	205	0	unrelated individuals
19	28	1	0	212	0	unrelated individuals
19	29	1	0	185	0	unrelated individuals
19	30	1	0	189	0	unrelated individuals
19	31	1	0	222	0	unrelated individuals
19	32	1	0	239	0	unrelated individuals
20	25	1	0	247	0	unrelated individuals
20	26	1	0	199	0	unrelated individuals
20	27	1	0	215	0	unrelated individuals
20	28	1	0	222	0	unrelated individuals
20	29	1	0	191	0	unrelated individuals
20	30	1	0	195	0	unrelated individuals
20	31	1	0	233	0	unrelated individuals
20	32	1	0	256	0	unrelated individuals
21	25	1	0	290	0	unrelated individuals
21	26	1	0	224	0	unrelated individuals

21	27	1	0	247	0	unrelated individuals
21	28	1	0	257	0	unrelated individuals
21	29	1	0	215	0	unrelated individuals
21	30	1	0	221	0	unrelated individuals
21	31	1	0	271	0	unrelated individuals
21	32	1	0	353	0	unrelated individuals
22	25	1	0	233	0	unrelated individuals
22	26	1	0	188	0	unrelated individuals
22	27	1	0	206	0	unrelated individuals
22	28	1	0	211	0	unrelated individuals
22	29	1	0	182	0	unrelated individuals
22	30	1	0	188	0	unrelated individuals
22	31	1	0	222	0	unrelated individuals
22	32	1	0	242	0	unrelated individuals
23	25	1	0	258	0	unrelated individuals
23	26	1	0	204	0	unrelated individuals
23	27	1	0	222	0	unrelated individuals
23	28	1	0	230	0	unrelated individuals
23	29	1	0	198	0	unrelated individuals
23	30	1	0	202	0	unrelated individuals
23	31	1	0	241	0	unrelated individuals
23	32	1	0	268	0	unrelated individuals
24	25	1	0	302	0	unrelated individuals
24	26	1	0	231	0	unrelated individuals
24	27	1	0	254	0	unrelated individuals
24	28	1	0	264	0	unrelated individuals
24	29	1	0	221	0	unrelated individuals
24	30	1	0	226	0	unrelated individuals
24	31	1	0	279	0	unrelated individuals
24	32	1	0	373	0	unrelated individuals
25	33	1	0	240	0	unrelated individuals
25	34	1	0	245	0	unrelated individuals
25	35	1	0	242	0	unrelated individuals
25	36	1	0	256	0	unrelated individuals
25	37	1	0	256	0	unrelated individuals
25	38	1	0	244	0	unrelated individuals
25	39	1	0	248	0	unrelated individuals
25	40	1	0	239	0	unrelated individuals
25	41	1	0	253	0	unrelated individuals
25	42	1	0	231	0	unrelated individuals
25	43	1	0	262	0	unrelated individuals

25	44	1	0	251	0	unrelated individuals
25	45	1	0	264	0	unrelated individuals
25	46	1	0	292	0	unrelated individuals
25	47	1	0	300	0	unrelated individuals
25	48	1	0	248	0	unrelated individuals
26	33	1	0	192	0	unrelated individuals
26	34	1	0	198	0	unrelated individuals
26	35	1	0	195	0	unrelated individuals
26	36	1	0	203	0	unrelated individuals
26	37	1	0	204	0	unrelated individuals
26	38	1	0	195	0	unrelated individuals
26	39	1	0	200	0	unrelated individuals
26	40	1	0	192	0	unrelated individuals
26	41	1	0	202	0	unrelated individuals
26	42	1	0	186	0	unrelated individuals
26	43	1	0	208	0	unrelated individuals
26	44	1	0	201	0	unrelated individuals
26	45	1	0	209	0	unrelated individuals
26	46	1	0	226	0	unrelated individuals
26	47	1	0	229	0	unrelated individuals
26	48	1	0	200	0	unrelated individuals
27	33	1	0	208	0	unrelated individuals
27	34	1	0	214	0	unrelated individuals
27	35	1	0	214	0	unrelated individuals
27	36	1	0	221	0	unrelated individuals
27	37	1	0	223	0	unrelated individuals
27	38	1	0	213	0	unrelated individuals
27	39	1	0	216	0	unrelated individuals
27	40	1	0	210	0	unrelated individuals
27	41	1	0	221	0	unrelated individuals
27	42	1	0	203	0	unrelated individuals
27	43	1	0	228	0	unrelated individuals
27	44	1	0	219	0	unrelated individuals
27	45	1	0	229	0	unrelated individuals
27	46	1	0	251	0	unrelated individuals
27	47	1	0	255	0	unrelated individuals
27	48	1	0	218	0	unrelated individuals
28	33	1	0	214	0	unrelated individuals
28	34	1	0	221	0	unrelated individuals
28	35	1	0	220	0	unrelated individuals
28	36	1	0	229	0	unrelated individuals

28	37	1	0	231	0	unrelated individuals
28	38	1	0	219	0	unrelated individuals
28	39	1	0	224	0	unrelated individuals
28	40	1	0	216	0	unrelated individuals
28	41	1	0	228	0	unrelated individuals
28	42	1	0	207	0	unrelated individuals
28	43	1	0	235	0	unrelated individuals
28	44	1	0	225	0	unrelated individuals
28	45	1	0	237	0	unrelated individuals
28	46	1	0	262	0	unrelated individuals
28	47	1	0	264	0	unrelated individuals
28	48	1	0	224	0	unrelated individuals
29	33	1	0	186	0	unrelated individuals
29	34	1	0	191	0	unrelated individuals
29	35	1	0	189	0	unrelated individuals
29	36	1	0	198	0	unrelated individuals
29	37	1	0	199	0	unrelated individuals
29	38	1	0	189	0	unrelated individuals
29	39	1	0	194	0	unrelated individuals
29	40	1	0	186	0	unrelated individuals
29	41	1	0	197	0	unrelated individuals
29	42	1	0	179	0	unrelated individuals
29	43	1	0	201	0	unrelated individuals
29	44	1	0	194	0	unrelated individuals
29	45	1	0	203	0	unrelated individuals
29	46	1	0	217	0	unrelated individuals
29	47	1	0	219	0	unrelated individuals
29	48	1	0	194	0	unrelated individuals
30	33	1	0	188	0	unrelated individuals
30	34	1	0	194	0	unrelated individuals
30	35	1	0	193	0	unrelated individuals
30	36	1	0	201	0	unrelated individuals
30	37	1	0	202	0	unrelated individuals
30	38	1	0	193	0	unrelated individuals
30	39	1	0	197	0	unrelated individuals
30	40	1	0	190	0	unrelated individuals
30	41	1	0	200	0	unrelated individuals
30	42	1	0	183	0	unrelated individuals
30	43	1	0	207	0	unrelated individuals
30	44	1	0	198	0	unrelated individuals
30	45	1	0	206	0	unrelated individuals

30	46	1	0	223	0	unrelated individuals
30	47	1	0	225	0	unrelated individuals
30	48	1	0	197	0	unrelated individuals
31	33	1	0	226	0	unrelated individuals
31	34	1	0	232	0	unrelated individuals
31	35	1	0	227	0	unrelated individuals
31	36	1	0	240	0	unrelated individuals
31	37	1	0	242	0	unrelated individuals
31	38	1	0	228	0	unrelated individuals
31	39	1	0	235	0	unrelated individuals
31	40	1	0	226	0	unrelated individuals
31	41	1	0	239	0	unrelated individuals
31	42	1	0	217	0	unrelated individuals
31	43	1	0	247	0	unrelated individuals
31	44	1	0	235	0	unrelated individuals
31	45	1	0	247	0	unrelated individuals
31	46	1	0	273	0	unrelated individuals
31	47	1	0	280	0	unrelated individuals
31	48	1	0	237	0	unrelated individuals
32	33	1	0	247	0	unrelated individuals
32	34	1	0	253	0	unrelated individuals
32	35	1	0	250	0	unrelated individuals
32	36	1	0	268	0	unrelated individuals
32	37	1	0	266	0	unrelated individuals
32	38	1	0	248	0	unrelated individuals
32	39	1	0	259	0	unrelated individuals
32	40	1	0	247	0	unrelated individuals
32	41	1	0	264	0	unrelated individuals
32	42	1	0	236	0	unrelated individuals
32	43	1	0	279	0	unrelated individuals
32	44	1	0	260	0	unrelated individuals
32	45	1	0	280	0	unrelated individuals
32	46	1	0	353	0	unrelated individuals
32	47	1	0	389	0	unrelated individuals
32	48	1	0	260	0	unrelated individuals
7	30	0,97457161	0,00010075	370	0,01268901	most distant kinship
7	27	0,96682636	0,00069197	1000	0,01641383	most distant kinship
5	30	0,96587645	0,00018111	761	0,0170165	most distant kinship
7	18	0,96486242	0,00015181	701	0,01753083	most distant kinship
7	28	0,96369334	0,00215635	1000	0,01761424	most distant kinship
5	18	0,9477719	0,0002431	827	0,02605328	most distant kinship

5	27	0,94562588	0,00077178	1000	0,02699411	most distant kinship
5	28	0,94014275	0,0026528	1000	0,02926543	most distant kinship
7	31	0,92791056	0,00083218	995	0,03583668	most distant kinship
5	31	0,90037797	0,00033486	884	0,0497273	most distant kinship
30	32	0,87505222	0,03240463	821	0,05437273	most distant kinship
7	25	0,83688355	0,10363882	452	0,05564852	most distant kinship
18	31	0,8821198	0,01020804	952	0,05638809	most distant kinship
18	29	0,87015787	0,02854727	683	0,05778425	most distant kinship
7	29	0,86319713	0,04183502	519	0,05794268	most distant kinship
18	30	0,87550109	0,01234596	905	0,05916297	most distant kinship
17	30	0,85256817	0,05209235	704	0,06069283	most distant kinship
29	32	0,83101412	0,07873612	574	0,06480891	most distant kinship
7	26	0,83641702	0,05695901	490	0,06755174	most distant kinship
7	32	0,83101454	0,06227814	536	0,06892319	most distant kinship
29	30	0,83891289	0,04536379	579	0,06920261	most distant kinship
5	25	0,81408887	0,09222369	505	0,06989964	most distant kinship
17	29	0,80699467	0,10156793	528	0,07111068	most distant kinship
18	26	0,831106	0,05167834	588	0,07152742	most distant kinship
5	29	0,82327008	0,06006945	492	0,0733476	most distant kinship
25	31	0,81964619	0,06633926	592	0,07359209	most distant kinship
7	17	0,80998843	0,07730139	505	0,07568044	most distant kinship
26	32	0,80604962	0,07199552	610	0,07897631	most distant kinship
27	32	0,8196004	0,03457274	863	0,08155661	most distant kinship
26	30	0,79931508	0,06525986	526	0,0840275	most distant kinship
18	27	0,81333147	0,03663324	696	0,08417595	most distant kinship
5	26	0,80105431	0,05795767	518	0,08498343	most distant kinship
29	31	0,79448797	0,06978291	516	0,08531029	most distant kinship
17	26	0,7807564	0,09302895	565	0,08636456	most distant kinship
5	32	0,80641553	0,03848782	675	0,08717028	most distant kinship
28	32	0,80680809	0,0322106	902	0,08854331	most distant kinship
17	27	0,79906014	0,04678347	786	0,08877406	most distant kinship
18	28	0,79684332	0,04729717	650	0,08975405	most distant kinship
27	29	0,76996266	0,08248717	502	0,09439687	most distant kinship
5	17	0,78172505	0,05774923	588	0,09470017	most distant kinship
30	31	0,75972381	0,09901074	477	0,09538541	most distant kinship
18	32	0,8015041	0,01506356	908	0,09548206	most distant kinship
18	25	0,74786582	0,11997579	477	0,09607314	most distant kinship
17	28	0,77852585	0,05507595	751	0,09696809	most distant kinship
28	29	0,75251108	0,09335137	487	0,10040662	most distant kinship
17	18	0,78246381	0,02721898	773	0,10196335	most distant kinship
25	30	0,74165201	0,10584395	473	0,10271301	most distant kinship

26	31	0,77161384	0,04341473	645	0,1033394	most distant kinship
26	27	0,73410956	0,08603753	518	0,11143583	most distant kinship
25	32	0,77030265	0,00246292	1000	0,11423295	most distant kinship
26	28	0,71535387	0,09452701	507	0,11869131	most distant kinship
31	32	0,73874352	0,04112117	680	0,12034795	most distant kinship
17	25	0,75473036	0,00586513	1000	0,12116854	most distant kinship
27	31	0,69299109	0,1101259	489	0,12597298	first kinship relationship
17	31	0,70942539	0,06198785	596	0,12979034	first kinship relationship
25	29	0,65458371	0,15326956	377	0,13439075	first kinship relationship
25	27	0,68007298	0,10191456	521	0,13448487	first kinship relationship
28	31	0,67028475	0,12017447	480	0,13481401	first kinship relationship
25	28	0,65990266	0,10614552	519	0,14351229	first kinship relationship
25	26	0,61978629	0,13770764	402	0,15567994	first kinship relationship
12	19	0,6688389	1,9297E-05	514	0,16557572	first kinship relationship
9	12	0,66761445	1,9751E-05	519	0,16618784	first kinship relationship
12	33	0,66545702	1,5357E-05	473	0,16726765	first kinship relationship
12	42	0,66048647	1,6957E-05	492	0,16975253	first kinship relationship
12	22	0,64494214	1,7763E-05	502	0,17752449	first kinship relationship
9	19	0,64258366	1,8406E-05	508	0,17870357	first kinship relationship
19	33	0,64104198	1,3995E-05	457	0,17947551	first kinship relationship
9	42	0,64095124	1,7073E-05	498	0,17952011	first kinship relationship
9	33	0,63979921	1,5061E-05	474	0,18009663	first kinship relationship
12	16	0,63566717	1,8548E-05	510	0,18216178	first kinship relationship
19	42	0,63226434	1,5921E-05	486	0,18386385	first kinship relationship
33	42	0,62882702	1,2464E-05	441	0,18558338	first kinship relationship
22	33	0,62105282	1,3684E-05	456	0,18947017	first kinship relationship
9	22	0,62046835	1,7819E-05	510	0,18976137	first kinship relationship
16	33	0,62027909	1,3403E-05	450	0,1898571	first kinship relationship
9	16	0,61904445	1,8793E-05	518	0,19047308	first kinship relationship
16	19	0,61789263	1,6793E-05	493	0,19104949	first kinship relationship
19	22	0,61088063	1,6793E-05	493	0,19455549	first kinship relationship
22	42	0,60972642	1,4421E-05	465	0,19513318	first kinship relationship
16	42	0,60782359	1,4421E-05	466	0,1960846	first kinship relationship
12	40	0,59536391	1,3089E-05	458	0,20231477	first kinship relationship
16	22	0,59370081	1,5611E-05	480	0,20314569	first kinship relationship
12	38	0,58000436	1,3098E-05	459	0,20999454	first kinship relationship
12	34	0,57676397	1,4577E-05	485	0,21161437	first kinship relationship
12	35	0,57101913	1,3338E-05	469	0,2144871	first kinship relationship
19	40	0,57074148	1,228E-05	452	0,21462619	first kinship relationship
9	40	0,56520488	1,2356E-05	457	0,21739447	first kinship relationship
19	38	0,56066919	1,1829E-05	453	0,21966245	first kinship relationship

9	38	0,56018775	1,2421E-05	461	0,21990302	first kinship relationship
12	20	0,55832851	1,3365E-05	470	0,2208324	first kinship relationship
12	39	0,55476996	1,3892E-05	478	0,22261155	first kinship relationship
9	34	0,55035371	1,3395E-05	471	0,2248198	first kinship relationship
40	42	0,55009019	1,0192E-05	422	0,22495236	first kinship relationship
19	34	0,54876551	1,2411E-05	460	0,22561414	first kinship relationship
12	44	0,54564189	1,4348E-05	486	0,22717547	first kinship relationship
12	37	0,54549094	1,3855E-05	481	0,22725107	first kinship relationship
19	35	0,54540691	1,1431E-05	447	0,22729369	first kinship relationship
9	35	0,54450364	1,2421E-05	462	0,22774507	first kinship relationship
33	40	0,54412416	9,6643E-06	416	0,22793551	first kinship relationship
38	42	0,54068346	9,7762E-06	416	0,22965583	first kinship relationship
12	48	0,54054761	1,3951E-05	481	0,22972271	first kinship relationship
22	40	0,5398294	1,1245E-05	445	0,23008249	first kinship relationship
9	20	0,53724948	1,2241E-05	462	0,2313722	first kinship relationship
12	36	0,53609102	1,4116E-05	491	0,23195096	first kinship relationship
16	40	0,53545472	1,085E-05	436	0,23226993	first kinship relationship
34	42	0,53477547	1,0529E-05	429	0,23260963	first kinship relationship
33	38	0,5346314	9,2378E-06	412	0,23268199	first kinship relationship
9	39	0,53331512	1,3249E-05	477	0,23333913	first kinship relationship
12	23	0,53127192	1,4879E-05	499	0,23436032	first kinship relationship
12	41	0,53095248	1,4428E-05	489	0,23452015	first kinship relationship
19	39	0,53046962	1,2239E-05	465	0,23476213	first kinship relationship
22	38	0,52926978	1,0713E-05	435	0,23536243	first kinship relationship
35	42	0,5290926	9,6523E-06	416	0,23545129	first kinship relationship
33	34	0,52835755	9,914E-06	423	0,23581875	first kinship relationship
19	20	0,52710003	1,177E-05	453	0,23644705	first kinship relationship
16	38	0,52552001	1,0628E-05	432	0,23723734	first kinship relationship
22	34	0,52328416	1,1535E-05	446	0,23835504	first kinship relationship
9	37	0,52239089	1,3068E-05	479	0,23880129	first kinship relationship
19	37	0,52223523	1,217E-05	464	0,23887934	first kinship relationship
9	44	0,52168625	1,3779E-05	489	0,23915343	first kinship relationship
20	33	0,52056479	9,4274E-06	416	0,23971525	first kinship relationship
33	35	0,52009518	9,1762E-06	409	0,23995012	first kinship relationship
19	44	0,51921249	1,2516E-05	468	0,24039063	first kinship relationship
16	34	0,51647211	1,0976E-05	439	0,2417612	first kinship relationship
20	42	0,51623919	9,7449E-06	420	0,24187797	first kinship relationship
12	15	0,51578733	1,4535E-05	502	0,2421027	first kinship relationship
19	48	0,51567802	1,2476E-05	468	0,24215787	first kinship relationship
9	48	0,51522126	1,338E-05	484	0,24238602	first kinship relationship
22	35	0,51443685	1,0431E-05	432	0,24277897	first kinship relationship

19	36	0,5130629	1,2076E-05	467	0,24346553	first kinship relationship
39	42	0,51251832	9,9535E-06	430	0,24373835	first kinship relationship
33	44	0,50960965	9,3239E-06	417	0,24519284	first kinship relationship
9	36	0,50937842	1,354E-05	493	0,2453074	first kinship relationship
12	43	0,50896799	1,5408E-05	514	0,24551215	first kinship relationship
16	35	0,50793619	1,0451E-05	436	0,24602929	first kinship relationship
9	23	0,50664456	1,4415E-05	499	0,24667411	first kinship relationship
33	39	0,50486617	9,834E-06	427	0,24756445	first kinship relationship
37	42	0,50452031	1,0033E-05	435	0,24773734	first kinship relationship
33	48	0,50412262	9,4238E-06	416	0,24793633	first kinship relationship
19	41	0,50304428	1,2264E-05	468	0,24847479	first kinship relationship
9	41	0,50298991	1,3549E-05	486	0,24850166	first kinship relationship
20	22	0,50202542	1,0676E-05	437	0,24898462	first kinship relationship
16	20	0,50160068	1,0443E-05	439	0,24919705	first kinship relationship
12	45	0,50143329	1,612E-05	523	0,24927932	first kinship relationship
22	39	0,49891968	1,0875E-05	442	0,25053744	first kinship relationship
19	23	0,49890418	1,3321E-05	481	0,25054458	first kinship relationship
33	37	0,49609625	9,3766E-06	422	0,25194953	first kinship relationship
23	33	0,49584545	9,747E-06	430	0,25207484	first kinship relationship
42	44	0,49505039	1,0223E-05	430	0,25247225	first kinship relationship
36	42	0,49456948	1,0048E-05	437	0,25271275	first kinship relationship
16	39	0,49402012	1,1139E-05	453	0,25298716	first kinship relationship
33	41	0,49382364	9,4052E-06	420	0,25308583	first kinship relationship
15	19	0,49288143	1,2741E-05	479	0,2535561	first kinship relationship
22	37	0,49283	1,0731E-05	449	0,25358232	first kinship relationship
42	48	0,48966559	1,0149E-05	436	0,25516467	first kinship relationship
23	42	0,48949039	1,0727E-05	445	0,25525212	first kinship relationship
9	15	0,48916757	1,339E-05	493	0,25541287	first kinship relationship
16	37	0,48832336	1,0772E-05	451	0,25583563	first kinship relationship
16	44	0,48793955	1,1291E-05	455	0,2560274	first kinship relationship
22	44	0,48787306	1,1313E-05	449	0,25606064	first kinship relationship
9	43	0,48568151	1,4078E-05	508	0,25715572	first kinship relationship
19	43	0,48560026	1,3114E-05	488	0,25719659	first kinship relationship
33	36	0,48545922	9,3383E-06	422	0,25726805	first kinship relationship
22	36	0,48301728	1,0941E-05	456	0,25848862	first kinship relationship
22	48	0,48254258	1,1233E-05	452	0,2587259	first kinship relationship
16	48	0,48126652	1,144E-05	458	0,25936388	first kinship relationship
15	33	0,47938457	9,5279E-06	429	0,26030533	first kinship relationship
12	14	0,47778051	1,9276E-05	575	0,26110493	first kinship relationship
12	13	0,47765606	1,8547E-05	565	0,26116734	first kinship relationship
16	36	0,47685193	1,1229E-05	459	0,26157123	first kinship relationship

9	45	0,47674368	1,4742E-05	520	0,26162447	first kinship relationship
41	42	0,47673663	9,9804E-06	442	0,26162919	first kinship relationship
15	42	0,47553995	1,0147E-05	442	0,26222749	first kinship relationship
19	45	0,47448036	1,3514E-05	495	0,26275644	first kinship relationship
22	41	0,47383136	1,0614E-05	447	0,26308166	first kinship relationship
16	23	0,47326284	1,18E-05	466	0,26336563	first kinship relationship
16	41	0,4716012	1,1005E-05	452	0,26419665	first kinship relationship
22	23	0,47053193	1,176E-05	464	0,2647311	first kinship relationship
33	43	0,46956048	9,4781E-06	432	0,26521739	first kinship relationship
15	22	0,4651673	1,1142E-05	462	0,26741357	first kinship relationship
33	45	0,46150965	9,7319E-06	443	0,26924274	first kinship relationship
42	43	0,45887409	1,0553E-05	456	0,27056032	first kinship relationship
13	19	0,45688961	1,5832E-05	535	0,27155124	first kinship relationship
12	46	0,4542083	2,2328E-05	614	0,27289027	first kinship relationship
22	43	0,45350388	1,1517E-05	468	0,27324518	first kinship relationship
15	16	0,45333048	1,0823E-05	455	0,27333205	first kinship relationship
14	19	0,4520927	1,6129E-05	540	0,27394962	first kinship relationship
12	24	0,45183698	2,5291E-05	646	0,27407519	first kinship relationship
42	45	0,45169392	1,0877E-05	467	0,27415032	first kinship relationship
12	21	0,45078158	2,5102E-05	644	0,27460294	first kinship relationship
9	13	0,45009751	1,7058E-05	563	0,27494698	first kinship relationship
16	43	0,44884951	1,1618E-05	475	0,27557234	first kinship relationship
9	14	0,44816142	1,6943E-05	555	0,27591505	first kinship relationship
12	47	0,44575946	2,6028E-05	655	0,27711376	first kinship relationship
22	45	0,44345615	1,1898E-05	478	0,27826895	first kinship relationship
16	45	0,44281227	1,1803E-05	474	0,27859091	first kinship relationship
13	33	0,44194643	1,089E-05	468	0,27902406	first kinship relationship
14	33	0,44114961	1,0954E-05	472	0,27942246	first kinship relationship
13	42	0,43730204	1,2006E-05	485	0,28134598	first kinship relationship
14	42	0,43525256	1,1948E-05	485	0,28237073	first kinship relationship
38	40	0,43413082	5,7846E-06	358	0,28293314	first kinship relationship
9	46	0,4286536	2,0024E-05	599	0,28566819	first kinship relationship
34	40	0,42713975	6,27E-06	367	0,28642856	first kinship relationship
9	24	0,42666597	2,1715E-05	624	0,28666159	first kinship relationship
19	46	0,42618938	1,8304E-05	577	0,28690073	first kinship relationship
9	21	0,4229011	2,225E-05	625	0,28854389	first kinship relationship
13	22	0,42280945	1,3173E-05	504	0,28859198	first kinship relationship
14	22	0,42209349	1,3602E-05	511	0,28894985	first kinship relationship
19	24	0,42149038	1,9997E-05	604	0,28924981	first kinship relationship
19	47	0,42081192	2,185E-05	617	0,28958858	first kinship relationship
19	21	0,42022525	2,0245E-05	600	0,28988232	first kinship relationship

34	38	0,41998883	5,9669E-06	365	0,29000409	first kinship relationship
20	40	0,41913487	5,5932E-06	350	0,29043117	first kinship relationship
9	47	0,41738399	2,3561E-05	644	0,29130212	first kinship relationship
35	40	0,41590738	5,6545E-06	352	0,2920449	first kinship relationship
13	16	0,41583949	1,3786E-05	520	0,29207681	first kinship relationship
33	46	0,41390445	1,218E-05	493	0,29304473	first kinship relationship
24	33	0,41315236	1,2742E-05	505	0,29342063	first kinship relationship
14	16	0,41272434	1,3529E-05	513	0,29363445	first kinship relationship
20	38	0,41221595	5,4344E-06	350	0,29389067	first kinship relationship
35	38	0,41128391	5,4675E-06	356	0,29435668	first kinship relationship
24	42	0,40728768	1,419E-05	532	0,29635261	first kinship relationship
21	33	0,40685476	1,2773E-05	507	0,29656943	first kinship relationship
33	47	0,40633058	1,3284E-05	515	0,29683139	first kinship relationship
20	34	0,40593927	5,8796E-06	367	0,29702889	first kinship relationship
40	44	0,40459093	5,6002E-06	359	0,29770313	first kinship relationship
21	42	0,40457297	1,4635E-05	537	0,29770986	first kinship relationship
5	7	0,32025583	0,16701151	299	0,29811921	first kinship relationship
42	46	0,40346293	1,3873E-05	526	0,29826506	first kinship relationship
34	35	0,40210463	5,778E-06	360	0,29894624	first kinship relationship
39	40	0,40102919	5,638E-06	356	0,29948399	first kinship relationship
40	48	0,39902	5,3988E-06	354	0,30048865	first kinship relationship
20	35	0,39652092	5,2665E-06	349	0,30173822	first kinship relationship
38	44	0,39522053	5,4618E-06	353	0,30238837	first kinship relationship
22	46	0,39396325	1,604E-05	550	0,30301436	first kinship relationship
42	47	0,39382919	1,5548E-05	554	0,30308152	first kinship relationship
38	39	0,3932737	5,5447E-06	358	0,30336176	first kinship relationship
37	40	0,39164703	5,5277E-06	362	0,3041751	first kinship relationship
16	46	0,39140815	1,5665E-05	552	0,30429201	first kinship relationship
23	40	0,39000834	5,6369E-06	362	0,30499442	first kinship relationship
16	24	0,38998581	1,7052E-05	572	0,30500283	first kinship relationship
34	44	0,38992157	5,8942E-06	370	0,30503774	first kinship relationship
38	48	0,38927323	5,2877E-06	359	0,30536206	first kinship relationship
34	39	0,388006	5,9464E-06	375	0,30599552	first kinship relationship
16	21	0,38721425	1,685E-05	563	0,30638866	first kinship relationship
22	47	0,38704252	1,8255E-05	586	0,30647417	first kinship relationship
22	24	0,38656196	1,6794E-05	570	0,30671482	first kinship relationship
37	38	0,38638039	5,2412E-06	359	0,30680849	first kinship relationship
40	41	0,38584358	5,3005E-06	356	0,30707688	first kinship relationship
21	22	0,3844479	1,7141E-05	573	0,30777176	first kinship relationship
34	48	0,38373926	5,8473E-06	371	0,30812891	first kinship relationship
35	44	0,38237547	5,3093E-06	354	0,30881094	first kinship relationship

20	39	0,38146328	5,1677E-06	355	0,30926707	first kinship relationship
23	38	0,38118728	5,3659E-06	359	0,30940502	first kinship relationship
16	47	0,38095724	1,8195E-05	584	0,30951683	first kinship relationship
35	39	0,37927043	5,2341E-06	353	0,31036348	first kinship relationship
36	40	0,37841423	5,3085E-06	359	0,31079156	first kinship relationship
34	37	0,37723366	5,6177E-06	369	0,31138177	first kinship relationship
38	41	0,37711747	5,0397E-06	352	0,31144001	first kinship relationship
23	34	0,37578658	5,8342E-06	371	0,31210525	first kinship relationship
35	48	0,37397593	5,0612E-06	354	0,31301077	first kinship relationship
34	41	0,37131592	5,4369E-06	362	0,31434068	first kinship relationship
15	40	0,37063355	5,1251E-06	351	0,31468194	first kinship relationship
36	38	0,36987666	4,9984E-06	351	0,31506042	first kinship relationship
20	37	0,3698101	4,9875E-06	345	0,3150937	first kinship relationship
20	44	0,36956323	5,1709E-06	348	0,31521709	first kinship relationship
39	44	0,36735349	5,4828E-06	366	0,31632189	first kinship relationship
35	37	0,36684156	4,9761E-06	352	0,31657798	first kinship relationship
23	35	0,36618636	5,0826E-06	351	0,31690555	first kinship relationship
34	36	0,36579444	5,496E-06	362	0,31710141	first kinship relationship
15	38	0,36417163	4,9208E-06	351	0,31791296	first kinship relationship
35	41	0,36255672	4,8933E-06	347	0,31872042	first kinship relationship
40	43	0,3622241	5,1508E-06	353	0,31888666	first kinship relationship
20	48	0,36190962	4,8636E-06	342	0,31904397	first kinship relationship
39	48	0,36013448	5,0783E-06	356	0,31993149	first kinship relationship
15	34	0,35861938	5,3841E-06	363	0,32068896	first kinship relationship
38	43	0,35731053	5,0028E-06	361	0,32134348	first kinship relationship
20	36	0,35693296	4,7477E-06	347	0,32153233	first kinship relationship
40	45	0,35563878	5,1396E-06	354	0,32217932	first kinship relationship
35	36	0,35505663	4,8447E-06	351	0,32247047	first kinship relationship
37	44	0,35457196	5,0156E-06	358	0,32271277	first kinship relationship
23	39	0,35273725	5,3325E-06	363	0,32363004	first kinship relationship
20	41	0,35110551	4,7368E-06	343	0,32444606	first kinship relationship
37	39	0,35109011	4,994E-06	354	0,3244537	first kinship relationship
34	43	0,35014858	5,3924E-06	366	0,32492436	first kinship relationship
20	23	0,34931586	4,9694E-06	351	0,32534083	first kinship relationship
37	48	0,34827469	4,9127E-06	357	0,32586142	first kinship relationship
39	41	0,34772308	4,744E-06	349	0,32613727	first kinship relationship
15	35	0,34655468	4,7217E-06	353	0,32672148	first kinship relationship
38	45	0,34623267	5,0261E-06	351	0,32688241	first kinship relationship
36	44	0,34217078	4,9068E-06	352	0,32891338	first kinship relationship
34	45	0,34077025	5,2776E-06	363	0,32961355	first kinship relationship
23	37	0,34034529	4,922E-06	355	0,32982613	first kinship relationship

36	39	0,33937403	4,6635E-06	350	0,33031182	first kinship relationship
35	43	0,33898412	4,5866E-06	346	0,33050679	first kinship relationship
26	29	0,27146734	0,13471959	298	0,33058643	first kinship relationship
44	48	0,33860074	5,0596E-06	351	0,33069836	first kinship relationship
23	44	0,33684639	5,2057E-06	358	0,3315755	first kinship relationship
36	48	0,33483849	4,6651E-06	348	0,33257959	first kinship relationship
15	20	0,33480596	4,4626E-06	341	0,3325959	first kinship relationship
37	41	0,3343949	4,698E-06	352	0,33280137	first kinship relationship
27	30	0,27651721	0,1156432	333	0,3328306	first kinship relationship
15	39	0,33189982	4,5543E-06	349	0,33404895	first kinship relationship
23	48	0,3305319	5,0045E-06	358	0,3347328	first kinship relationship
35	45	0,33011933	4,6536E-06	349	0,33493917	first kinship relationship
13	40	0,32953004	5,4342E-06	374	0,33523362	first kinship relationship
14	40	0,3276292	5,2462E-06	373	0,33618409	first kinship relationship
20	43	0,32701654	4,6878E-06	350	0,33649056	first kinship relationship
41	44	0,32625277	4,7943E-06	351	0,33687242	first kinship relationship
36	37	0,32605613	4,5611E-06	352	0,33697079	first kinship relationship
39	43	0,32595209	4,6736E-06	360	0,33702279	first kinship relationship
23	36	0,32588946	4,8003E-06	354	0,33705407	first kinship relationship
13	38	0,32282516	5,2149E-06	368	0,33858611	first kinship relationship
36	41	0,32215217	4,3501E-06	343	0,33892283	first kinship relationship
15	44	0,32119232	4,6352E-06	350	0,33940268	first kinship relationship
15	37	0,3198061	4,4373E-06	346	0,34009584	first kinship relationship
14	38	0,3192644	5,1403E-06	366	0,34036651	first kinship relationship
23	41	0,31914919	4,7284E-06	354	0,34042422	first kinship relationship
41	48	0,31830404	4,5757E-06	352	0,34084684	first kinship relationship
20	45	0,31697849	4,443E-06	346	0,34150964	first kinship relationship
13	34	0,31497679	5,6097E-06	382	0,3425102	first kinship relationship
39	45	0,31453552	4,6937E-06	355	0,34273107	first kinship relationship
14	34	0,31358015	5,7424E-06	389	0,34320849	first kinship relationship
15	48	0,31300241	4,3526E-06	340	0,34349771	first kinship relationship
37	43	0,31179724	4,4577E-06	352	0,34410026	first kinship relationship
28	30	0,24768627	0,12034252	347	0,34607123	first kinship relationship
15	36	0,30673561	4,1619E-06	341	0,34663115	first kinship relationship
13	35	0,30474468	4,7696E-06	363	0,34762647	first kinship relationship
15	23	0,30442535	4,5558E-06	350	0,34778619	first kinship relationship
14	35	0,30398323	4,769E-06	365	0,34800719	first kinship relationship
43	44	0,3034967	4,54E-06	353	0,34825051	first kinship relationship
37	45	0,30233155	4,3393E-06	347	0,34883314	first kinship relationship
40	46	0,30107787	5,5489E-06	379	0,34945968	first kinship relationship
15	41	0,29998098	4,1025E-06	330	0,35000848	first kinship relationship

36	43	0,29795398	4,0986E-06	344	0,35102199	first kinship relationship
43	48	0,29580702	4,2219E-06	348	0,35209544	first kinship relationship
23	43	0,29487695	4,6557E-06	354	0,35256036	first kinship relationship
24	40	0,29474758	5,6153E-06	390	0,35262481	first kinship relationship
13	20	0,29403303	4,5753E-06	354	0,35298234	first kinship relationship
44	45	0,29376998	4,4816E-06	354	0,35311389	first kinship relationship
38	46	0,29335157	5,2069E-06	378	0,35332291	first kinship relationship
14	20	0,29087213	4,7726E-06	358	0,35456274	first kinship relationship
40	47	0,29005361	5,7955E-06	388	0,35497175	first kinship relationship
21	40	0,28999828	5,6534E-06	382	0,35499945	first kinship relationship
24	38	0,28905364	5,4042E-06	386	0,35547183	first kinship relationship
13	39	0,28830435	4,7018E-06	363	0,35584665	first kinship relationship
36	45	0,28770145	3,9435E-06	336	0,35614829	first kinship relationship
14	39	0,28631204	4,7626E-06	367	0,35684279	first kinship relationship
45	48	0,2856069	4,2561E-06	345	0,35719548	first kinship relationship
23	45	0,28489169	4,5352E-06	352	0,35755302	first kinship relationship
34	46	0,28474275	5,8357E-06	392	0,35762717	first kinship relationship
21	38	0,28245787	5,5431E-06	387	0,35876968	first kinship relationship
24	34	0,28125915	6,0796E-06	411	0,35936891	first kinship relationship
38	47	0,28095826	5,695E-06	388	0,35951944	first kinship relationship
41	43	0,28086843	3,9791E-06	337	0,35956479	first kinship relationship
13	44	0,27768973	4,9031E-06	370	0,36115391	first kinship relationship
21	34	0,27663016	6,1179E-06	402	0,36168339	first kinship relationship
14	37	0,27576416	4,4085E-06	360	0,36211682	first kinship relationship
13	37	0,27558687	4,4237E-06	359	0,36220546	first kinship relationship
34	47	0,27523933	6,4918E-06	410	0,36237871	first kinship relationship
15	43	0,27483524	3,7583E-06	334	0,36258144	first kinship relationship
14	44	0,27481758	4,7496E-06	360	0,36259002	first kinship relationship
35	46	0,27452138	4,8986E-06	369	0,36273809	first kinship relationship
41	45	0,27416949	3,92E-06	339	0,36291427	first kinship relationship
13	48	0,27155243	4,5934E-06	361	0,36422264	first kinship relationship
24	35	0,27105034	4,9887E-06	373	0,36447358	first kinship relationship
14	48	0,26841262	4,4639E-06	353	0,36579258	first kinship relationship
15	45	0,26667548	3,697E-06	329	0,36666134	first kinship relationship
21	35	0,26597034	5,1782E-06	380	0,36701354	first kinship relationship
35	47	0,2632354	5,1109E-06	378	0,36838102	first kinship relationship
20	46	0,26268549	4,8228E-06	366	0,36865605	first kinship relationship
13	36	0,26230179	4,1728E-06	351	0,36884806	first kinship relationship
14	36	0,26108234	4,1058E-06	351	0,36945781	first kinship relationship
13	23	0,26070248	4,7256E-06	369	0,36964758	first kinship relationship
39	46	0,25930988	5,0111E-06	380	0,37034381	first kinship relationship

14	23	0,25825567	4,7009E-06	366	0,37087099	first kinship relationship
13	41	0,25688072	4,1944E-06	350	0,37155859	first kinship relationship
24	39	0,25520702	5,1578E-06	385	0,3723952	first kinship relationship
14	41	0,2544578	4,0755E-06	346	0,37277008	first kinship relationship
20	24	0,25396901	4,7724E-06	373	0,3730143	first kinship relationship
20	21	0,25133112	4,9306E-06	376	0,37433321	first kinship relationship
20	47	0,25084354	4,9428E-06	370	0,374577	first kinship relationship
21	39	0,24808544	4,9914E-06	383	0,37595603	first kinship relationship
39	47	0,24709759	5,2569E-06	387	0,37644989	first kinship relationship
43	45	0,24562076	3,5853E-06	334	0,37718872	first kinship relationship
37	46	0,24505745	4,5081E-06	366	0,37747015	first kinship relationship
24	44	0,24241477	5,0948E-06	383	0,37879134	first kinship relationship
24	37	0,23987701	4,5356E-06	370	0,38006036	first kinship relationship
44	46	0,23843784	4,7176E-06	371	0,3807799	first kinship relationship
21	44	0,23725858	5,0936E-06	376	0,38136943	first kinship relationship
13	15	0,2371519	3,7216E-06	337	0,38142312	first kinship relationship
24	48	0,23550207	4,6479E-06	376	0,38224781	first kinship relationship
21	37	0,23500983	4,7027E-06	374	0,38249391	first kinship relationship
37	47	0,23443868	4,6633E-06	371	0,38277949	first kinship relationship
14	15	0,2321992	3,7005E-06	335	0,38389948	first kinship relationship
36	46	0,23073785	4,0247E-06	353	0,38463007	first kinship relationship
21	48	0,23036914	4,7122E-06	375	0,38481425	first kinship relationship
46	48	0,22977283	4,305E-06	365	0,38511251	first kinship relationship
13	43	0,22963369	3,7407E-06	343	0,38518222	first kinship relationship
14	43	0,22760545	3,8042E-06	339	0,38619632	first kinship relationship
23	46	0,22617045	4,6814E-06	369	0,38691361	first kinship relationship
24	36	0,22588559	4,1768E-06	363	0,38705616	first kinship relationship
44	47	0,22445531	5,1442E-06	386	0,38777106	first kinship relationship
21	36	0,2203759	4,2257E-06	361	0,38981099	first kinship relationship
24	41	0,22003955	4,193E-06	357	0,38997917	first kinship relationship
36	47	0,21990404	4,3811E-06	361	0,39004689	first kinship relationship
23	24	0,21957834	4,821E-06	382	0,39020962	first kinship relationship
13	45	0,21957572	3,6353E-06	341	0,39021123	first kinship relationship
47	48	0,21799878	4,6747E-06	380	0,39099944	first kinship relationship
41	46	0,21706729	3,9327E-06	352	0,39146537	first kinship relationship
21	41	0,21705952	4,2334E-06	358	0,39146918	first kinship relationship
14	45	0,21699757	3,6179E-06	338	0,39150031	first kinship relationship
21	23	0,21633804	4,8498E-06	384	0,39182977	first kinship relationship
23	47	0,21414432	5,1271E-06	385	0,39292656	first kinship relationship
15	46	0,20716847	3,721E-06	341	0,39641483	first kinship relationship
41	47	0,20511525	4,2188E-06	361	0,39744132	first kinship relationship

15	24	0,20298396	3,8149E-06	347	0,39850707	first kinship relationship
15	21	0,19788565	3,7981E-06	348	0,40105622	genetically identical individuals
15	47	0,19439037	3,801E-06	343	0,40280386	genetically identical individuals
24	43	0,19328442	3,8797E-06	353	0,40335682	genetically identical individuals
43	46	0,18959667	3,6276E-06	344	0,40520076	genetically identical individuals
27	28	0,18527671	0,00654186	561	0,40572618	genetically identical individuals
21	43	0,18800581	3,7844E-06	346	0,40599615	genetically identical individuals
13	14	0,18598785	3,6018E-06	342	0,40700517	genetically identical individuals
24	45	0,1826204	3,727E-06	347	0,40868887	genetically identical individuals
45	46	0,18084573	3,4438E-06	338	0,40957628	genetically identical individuals
21	45	0,17875825	3,5944E-06	342	0,41061998	genetically identical individuals
43	47	0,17640606	3,8175E-06	356	0,41179601	genetically identical individuals
45	47	0,16743356	3,6507E-06	348	0,41628231	genetically identical individuals
13	46	0,15958725	3,6395E-06	352	0,42020546	genetically identical individuals
14	46	0,15557759	3,5707E-06	345	0,42221031	genetically identical individuals
13	24	0,15460375	3,8359E-06	361	0,42269716	genetically identical individuals
14	24	0,15192512	3,6858E-06	358	0,42403652	genetically identical individuals
14	21	0,14743278	3,8977E-06	355	0,42628264	genetically identical individuals
13	21	0,14736579	3,7455E-06	353	0,42631617	genetically identical individuals
13	47	0,14654471	4,031E-06	364	0,42672664	genetically identical individuals
14	47	0,14374409	4,0218E-06	369	0,42812695	genetically identical individuals
24	46	0,11701715	3,6721E-06	357	0,44149051	genetically identical individuals
17	32	0,11364522	0,0001226	624	0,44314674	genetically identical individuals
21	46	0,11235224	3,6441E-06	355	0,44382297	genetically identical individuals

21	24	0,10238532	3,5883E-06	354	0,44880644	genetically identical individuals
24	47	0,10185634	4,2241E-06	377	0,44907078	genetically identical individuals
46	47	0,09933372	3,9958E-06	371	0,45033214	genetically identical individuals
21	47	0,09742041	4,2341E-06	372	0,45128874	genetically identical individuals

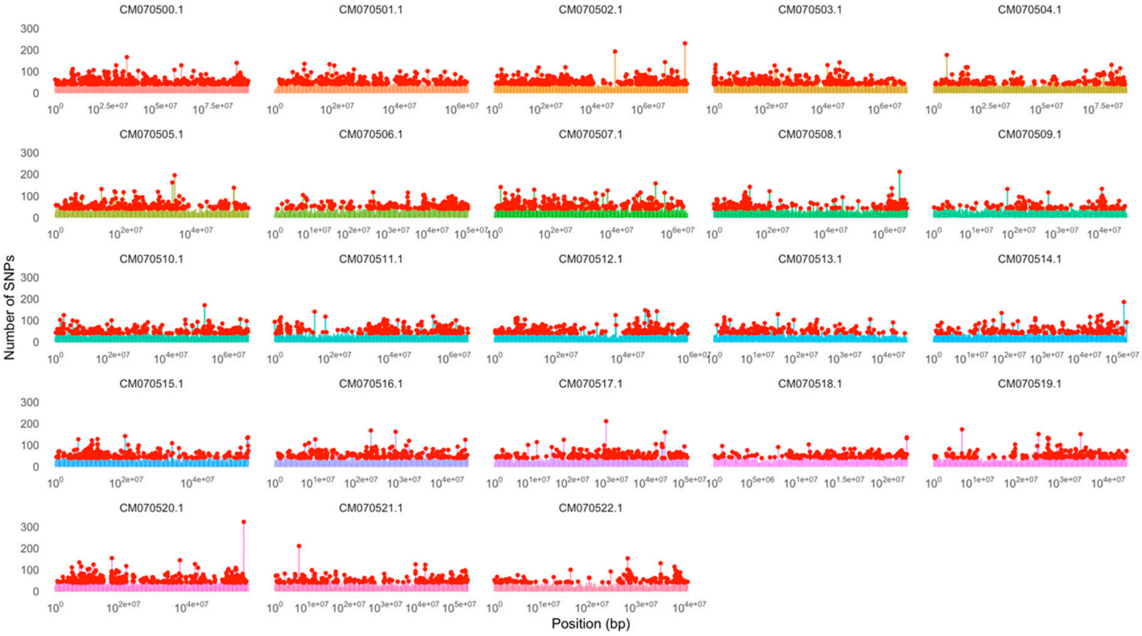

Figure S1. SNP density across the chromosomes of variants in *Guadua* angustifolia. SNP density was calculated using 10 kb sliding windows, revealing specific regions with notable variant accumulation, potentially associated with adaptive or diversification processes.
